# A deep learning algorithm to predict risk of pancreatic cancer from disease trajectories

**DOI:** 10.1038/s41591-023-02332-5

**Published:** 2023-05-08

**Authors:** Davide Placido, Bo Yuan, Jessica X. Hjaltelin, Chunlei Zheng, Amalie D. Haue, Piotr J. Chmura, Chen Yuan, Jihye Kim, Renato Umeton, Gregory Antell, Alexander Chowdhury, Alexandra Franz, Lauren Brais, Elizabeth Andrews, Debora S. Marks, Aviv Regev, Siamack Ayandeh, Mary T. Brophy, Nhan V. Do, Peter Kraft, Brian M. Wolpin, Michael H. Rosenthal, Nathanael R. Fillmore, Søren Brunak, Chris Sander

**Affiliations:** 1 Novo Nordisk Foundation Center for Protein Research, Faculty of Health and Medical Sciences, University of Copenhagen, Copenhagen, Denmark; 2 Harvard Medical School, Boston, MA USA; 3 Dana-Farber Cancer Institute, Boston, MA USA; 4 Broad Institute of MIT and Harvard, Boston, MA USA; 5 VA Boston Healthcare System, Boston, MA USA; 6 Boston University School of Medicine, Boston, MA USA; 7 Copenhagen University Hospital, Rigshospitalet, Copenhagen, Denmark; 8 Harvard T.H. Chan School of Public Health, Boston, MA USA; 9 Massachusetts Institute of Technology, Cambridge, MA USA; 10 Weill Cornell Medicine, New York City, NY USA; 11 Brigham and Women’s Hospital, Boston, MA USA; 12 Present Address: Genentech, Inc., South San Francisco, CA USA

**Keywords:** Cancer epidemiology, Machine learning, Cancer screening

## Abstract

Pancreatic cancer is an aggressive disease that typically presents late with poor outcomes, indicating a pronounced need for early detection. In this study, we applied artificial intelligence methods to clinical data from 6 million patients (24,000 pancreatic cancer cases) in Denmark (Danish National Patient Registry (DNPR)) and from 3 million patients (3,900 cases) in the United States (US Veterans Affairs (US-VA)). We trained machine learning models on the sequence of disease codes in clinical histories and tested prediction of cancer occurrence within incremental time windows (CancerRiskNet). For cancer occurrence within 36 months, the performance of the best DNPR model has area under the receiver operating characteristic (AUROC) curve = 0.88 and decreases to AUROC (3m) = 0.83 when disease events within 3 months before cancer diagnosis are excluded from training, with an estimated relative risk of 59 for 1,000 highest-risk patients older than age 50 years. Cross-application of the Danish model to US-VA data had lower performance (AUROC = 0.71), and retraining was needed to improve performance (AUROC = 0.78, AUROC (3m) = 0.76). These results improve the ability to design realistic surveillance programs for patients at elevated risk, potentially benefiting lifespan and quality of life by early detection of this aggressive cancer.

## Abstract (web summary)

A deep learning algorithm using electronic health records from two large cohorts of patients predicts the risk of pancreatic cancer from pre-cancer disease trajectories up to 3 years in advance, showing promising performance in retrospective validation.

## Main

Pancreatic cancer is a leading cause of cancer-related deaths worldwide, with increasing incidence^1^. Early diagnosis of pancreatic cancer is a key challenge, as the disease is typically detected at a late stage. Approximately 80% of patients with pancreatic cancer are diagnosed with locally advanced or distant metastatic disease, when long-term survival is extremely uncommon (2–9% of patients at 5 years)^2^. However, patients who present with early-stage disease can be cured by a combination of surgery, chemotherapy and radiotherapy. Thus, a better understanding of the risk factors for pancreatic cancer and detection at early stages has great potential to improve patient survival and reduce overall mortality.

The incidence of pancreatic cancer is substantially lower compared to other cancer types, such as lung, breast and colorectal cancer. Although it is true that age is a major risk factor, purely age-based population-wide screening for pancreatic cancer is impractical due to potentially costly clinical tests for a large number of patients with false-positive predictions. Moreover, few high-penetrance risk factors are known for pancreatic cancer, impeding early diagnosis of this disease. Risk of pancreatic cancer has been assessed for many years based on family history, behavioral and clinical risk factors and, more recently, circulating biomarkers and genetic predisposition^3–8^. Currently, some patients with high risk due to family history or rare inherited pathogenic variants or cystic lesions of the pancreas undergo serial pancreas-directed imaging to detect early pancreatic cancers. However, these patients account only for a small fraction of those who develop pancreatic cancer, and data on family history or genetic risk factors are often not available in the general population.

To address the challenge of early detection of pancreatic cancer in the general population^9,10^, we aimed to predict the risk of pancreatic cancer from real-world longitudinal clinical records of large numbers of patients and, among these, identify a moderate number of high-risk patients with the intent to facilitate the design of affordable surveillance programs for early detection. We can, thus, cast a wider net than the established successful surveillance programs in limited populations for which family history and/or germline genetic risk variants are available^11,12^.

The development of realistic risk prediction models requires a choice of appropriate machine learning (ML) methods, in particular deep learning techniques that work on large and noisy sequential datasets^13,14^. We build on earlier work in the field of risk assessment based on clinical data and disease trajectories using artificial intelligence (AI) methods^15,16^. AI methods have been applied to a number of clinical decision support problems^17^, such as choosing optimal time intervals for actions in intensive care units^18^, assessing cancer risk^19–22^ and predicting the risk of potentially acute disease progression, such as in kidney injury^23^, and the likelihood of a next diagnosis based on past electronic health record (EHR) sequences, in analogy to natural language processing^24,25^.

For risk assessment of pancreatic cancer, recently ML predictive models using patient records have been built using health interview survey data^26^, general practitioners’ health records controlled against patients with other cancer types^27^, real-world hospital system data^28,29^ and an EHR database provided by TriNetX, LLC^30,31^. Although demonstrating the information value of health records for cancer risk, these previous studies used only the occurrence of disease codes, not the time sequence of disease states in a patient trajectory. Previous studies used the Danish health registries to generate population-wide disease trajectories but in a descriptive manner^32,33^.

In this study, we exploited the power of recently developed ML technology by using information encoded in the time sequence of clinical events. This investigation was first carried out using the Danish National Patient Registry (DNPR), which contains data for 8.6 million patients from 1977 to 2018 (refs. ^34,35^), and subsequently for a smaller number of patients from the United States Veterans Affairs (US-VA) Corporate Data Warehouse (CDW). To optimize the extraction of predictive information from these records, we tested a diverse set of ML methods.

**Fig. 1:**
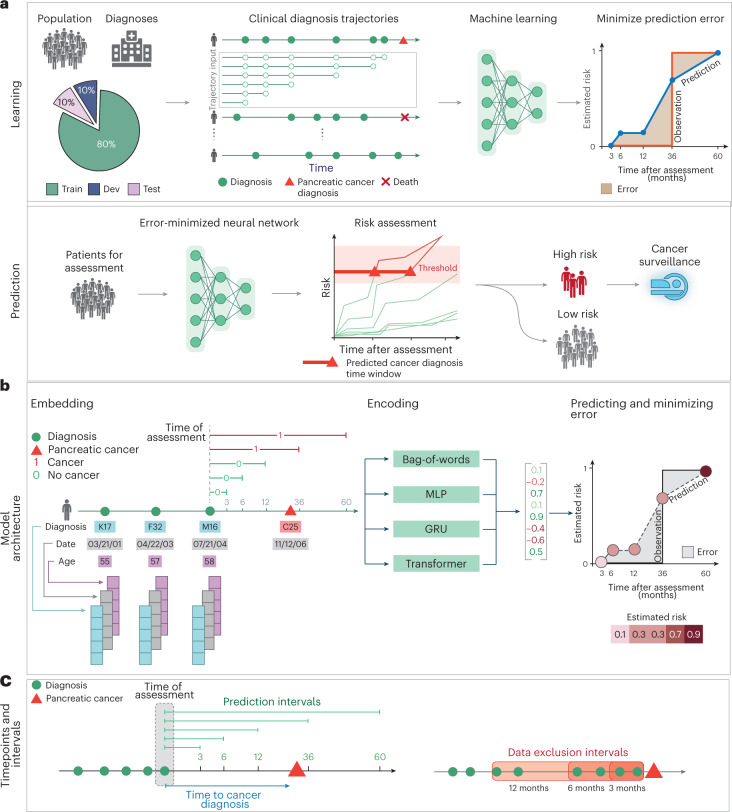
Training and prediction of pancreatic cancer risk from disease trajectories. **a**, Learning: The general ML workflow starts with partitioning the data into a training set (Train), a development set (Dev) and a test set (Test). The trajectories for training input are generated by sampling continuous subsequences of diagnoses for each patient’s diagnosis history, each starting with the first record but with different endpoints. The training and development sets are used for training so as to minimize the prediction error—that is, the difference between a risk score function (prediction) and a step function (observation), summed over all instances. Prediction: A model’s ability to accurately predict is evaluated using the withheld test set. The prediction model, depending on the prediction threshold selected from among possible operational points, discriminates between patients at higher and lower risk of pancreatic cancer. The risk model can guide the development of surveillance initiatives. **b**, The model trained with real-world clinical data has three steps: embedding, encoding and prediction. The embedding machine transforms categorical disease codes and timestamps of these disease codes into a lower-dimensional real number continuous space. The encoding machine extracts information from a disease history and summarizes each sequence in a characteristic fingerprint in the latent space (vertical vector). The prediction machine then uses the fingerprint to generate predictions for cancer occurrence within different time intervals after the time of assessment (3, 6, 12, 36 and 60 months). The model parameters are trained by minimizing the difference between the predicted and the observed cancer occurrence. **c**, Terminology for timepoints and intervals. The last event of a disease trajectory coincides with the time of assessment. From the time of assessment, cancer risk is assessed within 3, 6, 12, 36 and 60 months. To test the influence of close-to-cancer diagnosis codes on the prediction of cancer occurrence, exclusion intervals are used to remove diagnoses in the last 3, 6 and 12 months before cancer diagnosis.

The likely action resulting from a personalized positive prediction of cancer risk ideally should take into account the probability of the disease occurring within a shorter or longer timeframe (Fig. 1). We, therefore, designed the AI method not only to predict whether cancer is likely to occur but also to provide risk assessment in incremental time intervals after the predictive assessment of risk, following earlier work on mammography-based breast cancer risk prediction^20^. To facilitate interpretation of what the trained models have learned, we analyzed which diagnoses in a patient’s history of diagnosis codes are flagged by the method as most informative of cancer risk; and we propose a practical scenario for surveillance programs, taking into consideration the availability of real-world data, the estimated accuracy of prediction on such data, the scope of a surveillance program, the likely cost and success rate of surveillance methods and the overall potential benefit of early treatment (Supplementary Notes).

## Results

### Datasets

**Fig. 2:**
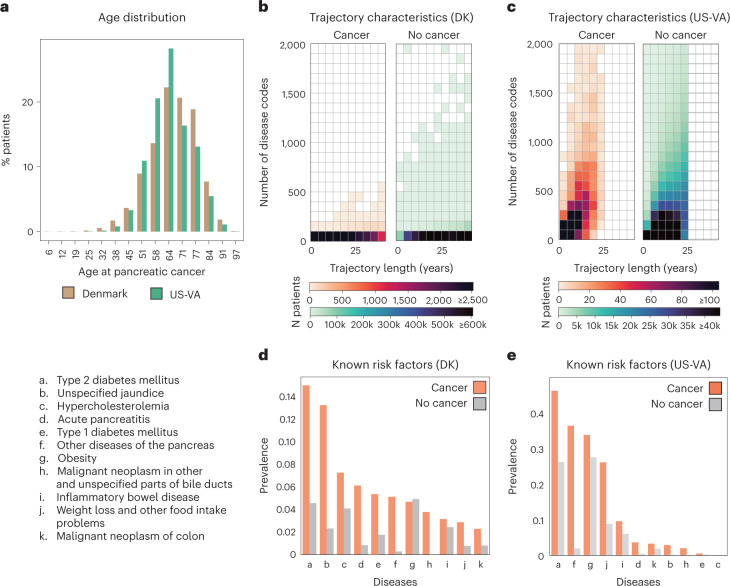
Characteristics of the Danish and US-VA patient registries. **a**, Distributions for age at pancreatic cancer diagnosis in the two cohorts. **b**,**c**, The Danish (DK) dataset has a longer median length of disease trajectories but lower median number of disease codes per patient compared to the US-VA dataset, so the ML process, independently in each dataset, has to cope with very different distributions of disease trajectories in terms of length of trajectories and density of the number of disease codes. Color level indicates the number of patients in a given bin. **d**,**e**, Background check on the distribution disease codes in the clinical records: prevalence of known risk factors in cancer versus non-cancer patients in the DK (**d**) and US-VA (**e**) datasets, counting whether a disease code occurred at least once in a patient’s history previous to their pancreatic cancer code (cancer) or 2 years previous to the end of data (no cancer).

**Extended Data Fig. 1:**
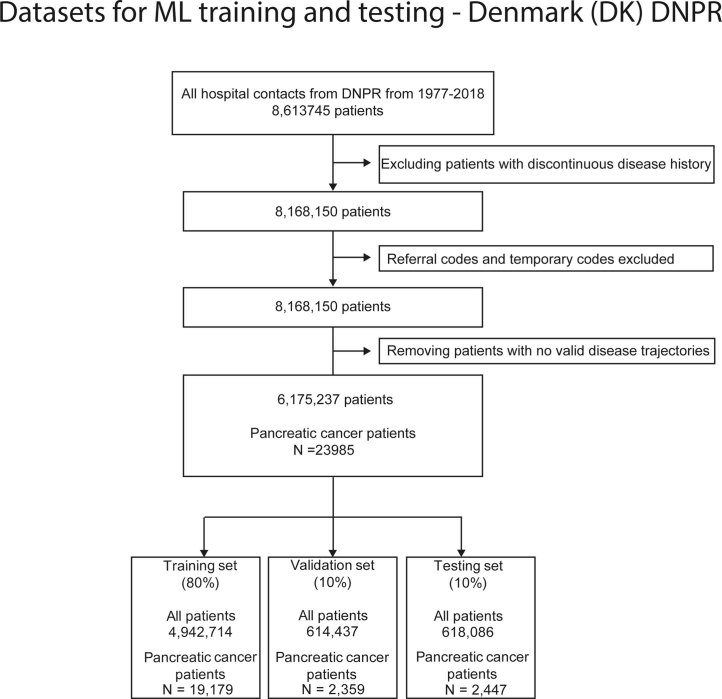
Preprocessing and filtering of the DK disease trajectory datasets. Filtering of the Danish (DK-DNPR) patient registries prior to training. In the Danish dataset, patient status codes were used to remove discontinuous disease histories such as patients living in Greenland, patients with alterations in their patient ID or patients who lack a stable residence in Denmark. We also removed referral and temporary diagnosis codes which are not the final diagnosis codes and can be misleading to use for training. Patients with short trajectories (<5 diagnosis codes) were removed. The final set of patients were split into Training (80 %), Validation (10%) and Testing set (10%).

**Extended Data Fig. 2:**
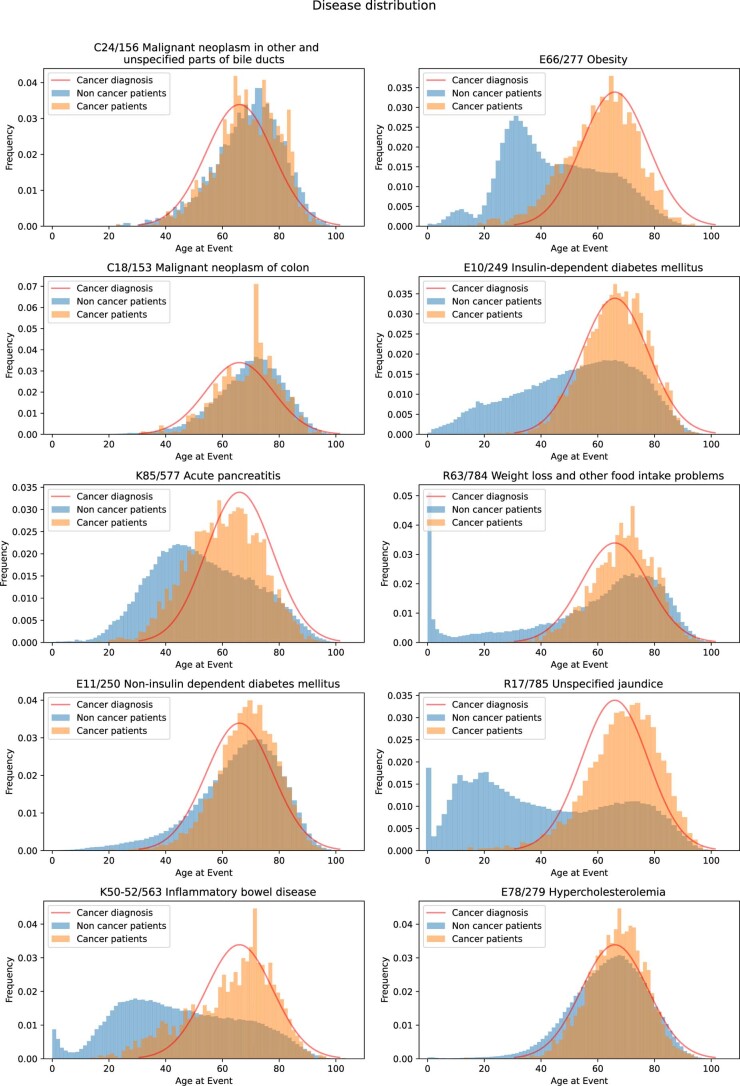
Distribution of disease codes as a function of age in the DNPR (Denmark) database. Distribution of disease codes for a representative subset of diseases known to contribute to the risk of pancreatic cancer, as a fraction of all pancreatic cancer patients (orange) and all non-cancer patients (blue). The similarity of the distributions for some of these diseases with the distribution of occurrence of pancreatic cancer (red line, Gaussian fit to cancer diagnosis data) is consistent with either a direct or indirect contribution to cancer risk - but not taken as evidence in this work. The disease codes are ICD-10/ICD-8.

We used disease trajectories from the DNPR, with demographic information from the Central Person Registry (CPR)^36^. DNPR covers approximately 8.6 million patients with 229 million hospital diagnoses, with, on average, 26.7 diagnosis codes per patient. For training, we used trajectories of International Classification of Diseases (ICD) diagnostic codes, down to the three-character category in the ICD hierarchy, with explicit timestamps for each hospital contact from January 1977 to April 2018, for a total of 6.2 million patients after standard filtering (Methods), including 23,985 pancreatic cancer cases (Fig. 2a,b,d, Table 1, Extended Data Figs. 1 and 2 and Supplementary Table 1).

**Table 1:** Characteristics of the Danish and US-VA datasets

General cohort information	Danish dataset	US-VA dataset
Dataset timeline	1977–2018	1999–2020
Total *n* patients	8,110,706	2,962,383
Male (%)	4,030,504 (49.7%)	2,538,762 (85.7%)
Female (%)	4,080,202 (50.3%)	423,621 (14.3%)
Median *n* disease codes per patient	22	188
Median length of trajectory in years	23.0	12.0
**PC cohort information**		
Total *n* patients	23,985	3,869
Male (%)	11,880 (49.5%)	3,741 (96.7%)
Female (%)	12,105 (50.5%)	128 (3.3%)
Median *n* disease codes per patient	18	121
Median length of trajectory in years	17.0	8.0
Median age at PC diagnosis	70.0	68.0
*n* disease codes 0–3 months pre-PC	95,358	125,305
*n* disease codes 3–6 months pre-PC	27,131	56,198
*n* disease codes 6–12 months pre-PC	38,109	97,911
*n* disease codes >12 months pre-PC	480,830	1,188,199

PC, pancreatic cancer.

**Extended Data Fig. 3:**
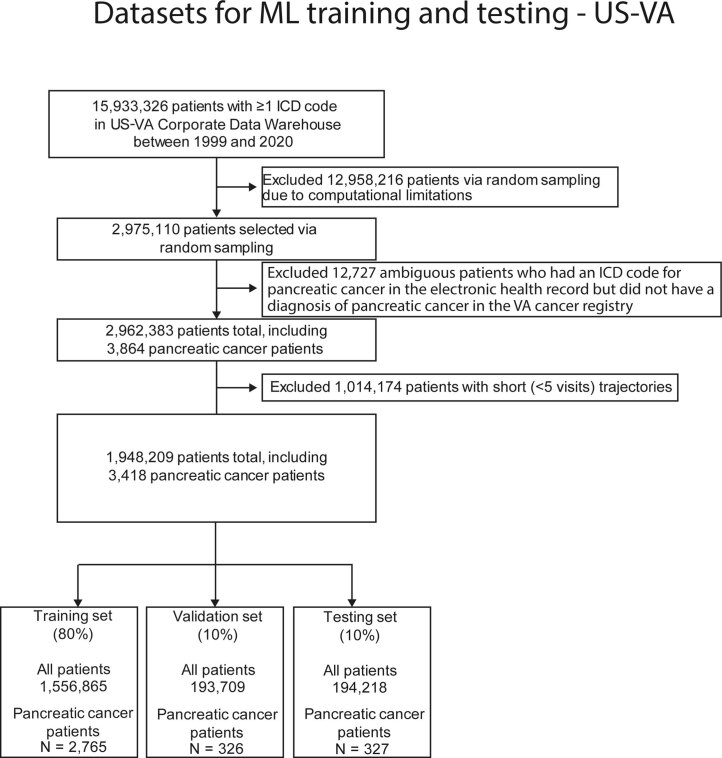
Preprocessing and filtering of US-VA disease trajectory datasets. Filtering of the US-VA patient registries prior to training. For the US-VA dataset, around 3 million of patients were randomly sampled due to computational limitations and patients with ICD-9/10 for pancreatic cancer, but without entries in US-VA cancer registry were excluded. Similar to the Danish dataset filtering, short trajectories (<5 diagnosis codes) were removed and patients were split into Training (80 %), Validation (10%) and Test set (10%).

For validation in another healthcare system, we similarly used longitudinal clinical records from 1999 to 2020 from the US-VA CDW (data warehouse), which integrates both EHRs and cancer registry data nationwide (Fig. 2a,c,e). For training, we used trajectories from a selected dataset (Methods) with a total of 3.0 million patients, including 3,864 pancreatic cancer cases (Table 1 and Extended Data Fig. 3). On average, the health records in the US-VA dataset have shorter (median 12 years in US-VA versus 23 years in DNPR) but substantially denser disease histories (median 188 records per patient in US-VA versus 22 records per patient in DNPR). These differences likely reflect the differences in population (entire population in Denmark versus military veterans in the US-VA) and in healthcare system practices, such as referral, documentation and billing.

### ML model architecture

The ML model for predicting cancer risk from disease trajectories consists of (1) input data for each event in a trajectory (diagnosis code and timestamps); (2) embedding of the event features onto real number vectors; (3) encoding the trajectories in a lower-dimensional latent space; and (4) predicting time-dependent cancer risk (Methods). The longitudinal nature of the disease trajectories allows us to construct time sequence models using sequential neural networks, such as gated recurrent unit (GRU) models^37^ and the Transformer model^38^. As computational control, we also tested a bag-of-words (that is, bag-of-disease-codes) approach that ignores the time and order of disease events. Each of the models learns to estimate the risk of cancer within distinct prediction intervals ending 3, 6, 12, 36 or 60 months after the end of a trajectory (the time of risk assessment) rather than just binary (yes or no) prediction of risk that cancer will occur at any time after assessment.

To avoid overfitting and to test generalizability of model predictions, we partitioned patient records randomly into 80%/10%/10% training/development/test sets. We conducted training only on the training set and used the development set to examine the performance for different hyperparameter settings, which guides model selection. The performance of the selected models was evaluated on the fully withheld test set of trajectories and reported as an estimate of performance in prospective patients. To test the influence of close-to-cancer diagnosis codes on the prediction of cancer occurrence, in training we also removed from input diagnoses in the last 3, 6 and 12 months before cancer diagnosis.

### Evaluation of model performance

**Fig. 3:**
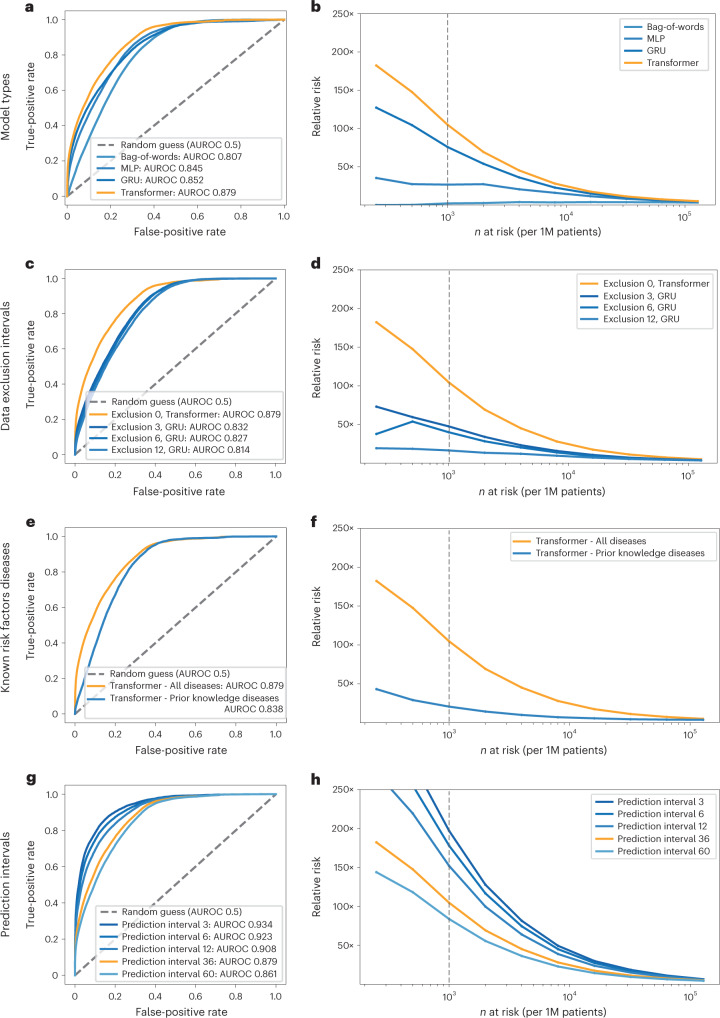
Performance of the ML model on clinical record trajectories in predicting pancreatic cancer occurrence in the Danish dataset. For each model and prediction evaluation, performance is better for larger AUROC (**a**,**c**,**e**,**g**) and for higher RR (Relative risk) for the *n* (horizontal axis) highest-risk patients (**b,d,f,h**). **a**,**b**, Choice of algorithm: The Transformer algorithm is best with AUROC = 0.879 (no data exclusion, 36-month prediction interval). **c**,**d**, Choice of input data: Prediction performance declines with exclusion interval, in training, of *k* = 3, 6 and 12 months of data between the end of a disease trajectory and cancer occurrence (best model for each exclusion interval, for 36-month prediction interval). **e**,**f**, Choice of input data: Prediction is better for all 2,000 ICD level-3 disease codes used throughout in training (Methods) compared to only the subset of 23 known risk factors, using a Transformer, all data (Exclusion 0), for the 36-month prediction interval. **g**,**h**, Choice of prediction task: Prediction of cancer is more difficult for larger prediction intervals, the time interval within which cancer is predicted to occur after assessment (Transformer model, all data). We reported prediction performance for the 36-month prediction interval (orange in **g** and **h**) in the above panels (**a-f**), as this is a reasonable choice for design of a surveillance program in clinical practice. **b**,**d**,**f**,**h**, Prediction performance at a particular operational point—for example (**d**), for *n* = 1,000 highest-risk patients (vertical dotted line) out of 1 million (1M) patients, the RR is 104.7 for the 36-month prediction interval using all data and 47.6 with 3-month data exclusion.

We evaluated the prediction performance of the different models trained in the DNPR using the area under the receiver operating characteristic (AUROC) and relative risk (RR) curves (Fig. 3). All performance metrics are calculated on the basis of applying each trained risk assessment model to the test set. The test set is strictly withheld during training and hyperparameter search. In the final performance evaluation of different types of ML models on the test sets, the models, which explicitly use and encode the time sequence of disease codes—that is, GRU and Transformer—ranked highest by AUROC (Fig. 3a,b and Supplementary Table 3). For the prediction of cancer incidence within 3 years of the assessment date (the date of risk prediction), the Transformer model had the best performance (AUROC = 0.879 (0.877–0.880)) followed by GRU (AUROC = 0.852 (0.850–0.854)).

**Fig. 4:**
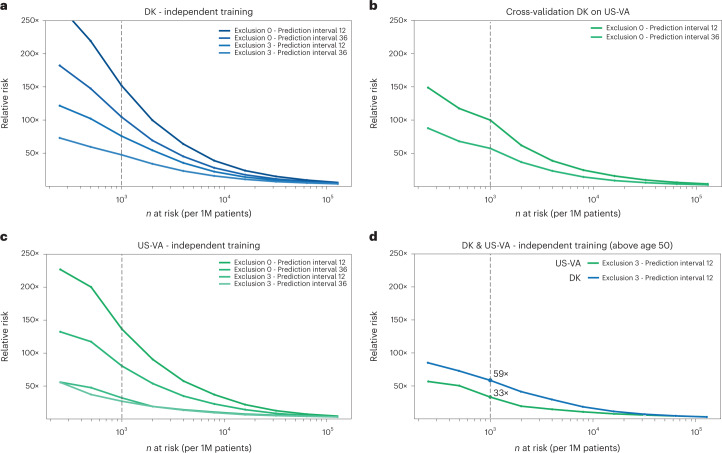
Estimated performance of a surveillance program for high-risk patients in different health systems and with different operational choices. Estimated relative risk (RR) for the top *n* (horizontal axis) high-risk patients is based on evaluating the accuracy of prediction on the withheld test set (**a**,**c**,**d**) and on a full external dataset (**b**). **a**,**c**, In designing surveillance programs, one can choose between models trained on all data (Exclusion 0) versus models trained excluding data from the last 3 months before cancer occurrence (Exclusion 3) and between prediction for cancer within 12 months or 36 months of assessment (legend top right in each panel). **b**, Estimated performance is somewhat lower for cross-application of a model trained on Danish (DK) data applied to US-VA patient data, illustrating the challenge of deriving globally valid prediction tools without independent localized or system-specific training. **d**, A proposed practical choice for a surveillance program with good estimated accuracy of prediction, in either system, would involve application of independently trained models with 3-month data exclusion for a prediction interval of 12 months for patients older than age 50 years. 1M, 1 million.

To gain a better intuition regarding the impact of applying a model in a real case scenario, we also report the RR score of patients with cancer in the high-risk group as predicted by the ML model (Figs. 3b,d,f,h and 4). The RR score is defined at a given operational decision point (Methods). This assesses by what factor a prediction method does better than a random model. The RR for the 36-month prediction interval is 104.7 for the Transformer model (with time sequences), at an operational point defined by the *n* = 1,000 highest-risk patients out of 1 million patients (0.1% at highest risk; notation: N1000).

Earlier work also developed ML methods on real-world clinical records to predict pancreatic cancer risk^28–31^. These previous studies had encouraging results, but neither used the time sequence of disease histories to extract time-sequential longitudinal features. For comparison, we implemented analogous approaches, a bag-of-words model and a multilayer perceptron (MLP) model. We evaluated the non-time-sequential models on the DNPR dataset, and the performance for predicting cancer occurrence within 36 months was AUROC = 0.807 (0.805–0.809) for the bag-of-words model and AUROC = 0.845 (0.843–0.847) for the MLP model (Fig. 3a). The RR was also much lower (2.1 and 26.6, respectively) compared to that of time-sequential models (for example, 104.7 for Transformer).

### Performance with data exclusion

Disease codes within a very short time before diagnosis of pancreatic cancer are most probably directly indicative such that, even without any ML, well-trained clinicians would include pancreatic cancer at a high rank in their differential diagnosis. Disease codes just before pancreatic cancer occurrence may also indirectly cover pancreatic cancer (for example, neoplasm of the digestive tract) and, thus, reflect the label one wants to infer. To reduce undue influence of these disease codes in training, we separately trained the models excluding input diseases diagnoses from the last 3, 6 and 12 months before the diagnosis of pancreatic cancer (‘data exclusion’). As expected, when training with data exclusion, the performance decreased from AUROC = 0.879 to AUROC = 0.843/0.829/0.827 for 3-/6-/12-month data exclusion for the best models, all for prediction of cancer occurrence within 36 months (DNPR dataset; Fig. 3c).

### Performance for training on known risk factors

We explored the right level of granularity and completeness of disease codes to train on. One can train on a smaller set of disease codes that occur in patient disease trajectories. For example, one can use prior knowledge and limit the input for training to known risk factors—that is, diseases that have been reported to be indicative of the likely occurrence of pancreatic cancer^11,39^. We found that prediction performance with the subset of ICD codes for 23 known risk factors reduces prediction accuracy of the Transformer model to AUROC = 0.838 compared to AUROC = 0.879 for all diagnosis codes, and, therefore, we used the latter (ICD level-3, 2,000 disease codes) throughout the rest of the work (Fig. 3e,f and Supplementary Table 4).

### Prediction for time intervals

**Fig. 5:**
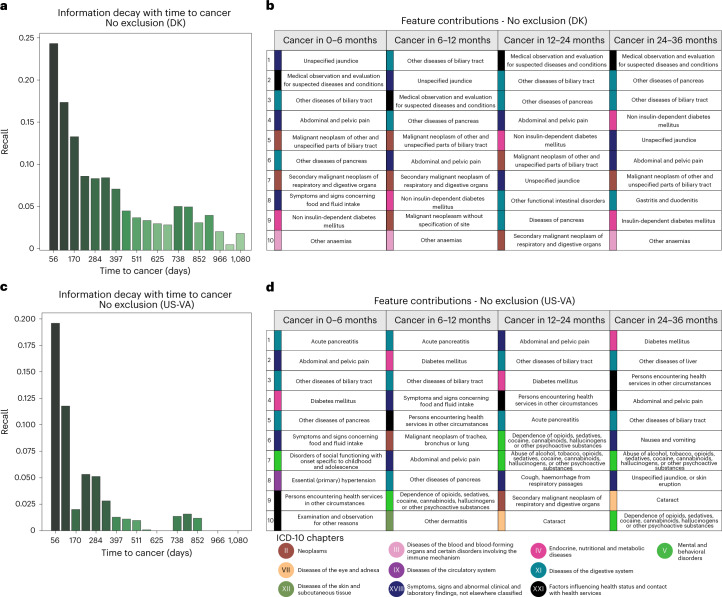
Predictive capacity and feature contributions of disease trajectories. **a**,**c**, Distribution of recall (sensitivity) values at the F1 operational point (Methods) as a function of time to cancer (time between the end of a disease trajectory and cancer diagnosis). As expected, recall levels decrease with longer time to cancer, from 8% for cancer occurring about 1 year after assessment to a recall of 4% for cancer occurring about 3 years after assessment (DNPR). This suggests that the model learns not only from symptoms very close to pancreatic cancer but also from longer disease histories, albeit at lower accuracy. **a**, Danish system (DK), for models trained on all data (no data exclusion). **c**, US-VA system, for models trained on all data. **b**,**d**, Top 10 features that contribute to the cancer prediction in time-to-cancer intervals of 0–6, 6–12, 12–24 and 24–36 months for the Danish (DK) (**b**) and US-VA (**d**) systems. The features are sorted by the contribution score (Supplementary Table 5). We used an integrated gradients (IG) method to calculate the contribution score for each input feature for each trajectory and then summed over all trajectories with cancer diagnosis within the indicated time interval.

It is of particular clinical interest to consider the risk of cancer over different time intervals. The ML models in this work are designed to report risk scores for pancreatic cancer occurrence within 3, 6, 12, 36 and 60 months of the date of risk assessment. As expected, it is more challenging to predict cancer occurrence within longer rather than shorter time intervals, as the longer time intervals allow for larger time gaps between the end of the disease trajectory (time of assessment) and the time of cancer diagnosis (Fig. 5a,b). Indeed, prediction performance for the Transformer model decreases from an AUROC of 0.908 (0.906–0.911) for cancer occurrence within 12 months to an AUROC of 0.879 (0.877–0.880) for occurrence within 3 years (without data exclusion) (Fig. 3g,h).

### Application in a different healthcare system

To assess the predictive performance of the model in other healthcare systems, we applied the best ML model trained on the Danish dataset to disease trajectories of patients in the US-VA dataset, without any adaptation except for mapping the ICD codes from one system to the other. Prediction performance for cancer occurrence within 36 months after assessment declined from an AUROC of 0.879 (0.877–0.880) and RR = 104.6, for a Denmark-trained Transformer model applied to DNPR patient data (test set), to an AUROC of 0.710 (0.708–0.712) and RR = 57.4, for the same model applied to US-VA patient data (Fig. 4b). The most striking difference in input data between the two systems is the shorter and more dense disease history in the US-VA trajectories compared to the Danish ones (Fig. 2b,c).

Motivated by the decrease in performance when testing the Denmark-derived model on the US-VA dataset, we trained and evaluated the model on the US-VA dataset from scratch. For the independently trained model, the performance is clearly higher than in cross-application, with a test set AUROC of 0.775 (0.772–0.778) and RR = 80.4 at the N1000 operational point (0.1% at highest risk) for cancer occurring within 36 months (Fig. 4c). The difference in performance of the independently trained models in the two healthcare systems may be in part due to differences in medical and reporting practices or in demographics, including different age and sex distributions.

### Estimated performance of a realistic surveillance program

A realistic surveillance program has to use an operational point (decision threshold) taking into consideration cost and benefit in real-world clinical practice. Although we have no empirical data on cost and benefit in this study, we can evaluate predictive performance, which is a key factor in assessing benefit. An example (Fig. 4d), for a potentially realistic choice of model and decision threshold, is a surveillance program at an operational point of *n* = 1,000 (0.1%) highest-risk patients out of 1 million patients—that is, at a risk fraction of 0.1% of all patients assessed. At this point, the best models trained with 3 months of data exclusion evaluated for the 12-month prediction interval for patients older than age 50 years obtain a relative risk of RR = 58.7 for DNPR (GRU model) and of RR = 33.1 for US-VA (Transformer model). Corresponding models using all the data (no exclusion) have higher values of RR. Considerations for the design of a surveillance program are further discussed below.

### Predictive features

**Extended Data Fig. 4:**
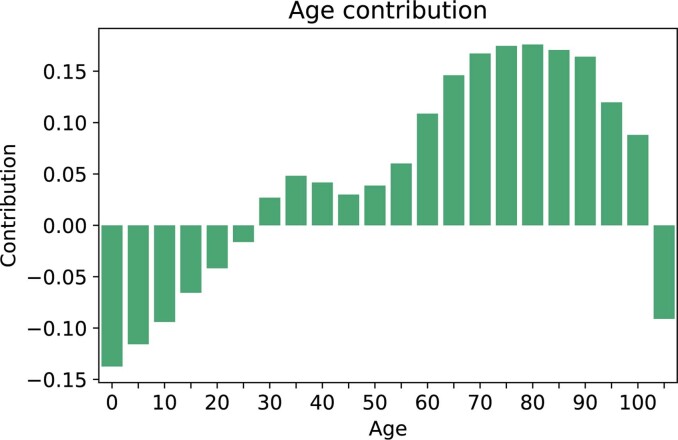
Age as a contributing factor. The integrated gradient method was used to extract the contribution (arbitrary units) of patient age to the prediction at the time of assessment. This confirmed that the positive contribution to risk rises strongly from age 50. As for the disease contributions, the age contribution was calculated in relation to the 3 year (after the time of assessment/prediction) cancer risk.

Although the principal criterion for the potential impact of prediction–surveillance programs is robust predictive performance, it is of interest to interpret the features of any predictive method: which diagnoses are most informative of cancer risk? Computational methods can infer the contribution of a particular input variable to the prediction by an ML engine—for example, the integrated gradient (IG) algorithm^40^ (Fig. 5c,d, Extended Data Fig. 4 and Supplementary Table 5). The IG contribution was calculated separately for different times between assessment and cancer diagnosis, in particular at 0–6 months, 6–12 months, 12–24 months and 24–36 months after assessment, for all patients who developed cancer. As expected, there is a tendency for codes, which, in normal clinical practice, are known to indicate the potential presence of pancreatic cancer, to have a higher contribution to prediction for trajectories that end closer to cancer diagnosis. On the other hand, putative early risk factors have a higher IG score for trajectories that end many months before cancer diagnosis. Although there is not necessarily a causative relation between a single predictive feature and outcome, this approach provides useful insight on the correlation between specific diagnoses and pancreatic cancer.

The top contributing features extracted from the trajectories with time to cancer diagnosis in 0–6 months—such as unspecified jaundice, diseases of biliary tract, abdominal and pelvic pain, weight loss and neoplasms of digestive organs—may be symptoms of, or otherwise closely related to, pancreatic cancer (Supplementary Table 5a). It is also of interest to identify early risk factors for pancreatic cancer. For trajectories with longer time between assessment and cancer diagnosis, other disease codes, such as type 2 diabetes and insulin-independent diabetes, make an increasingly large contribution, consistent with epidemiological studies^8,39,41,42^ and the observed disease distribution in the DNPR and US-VA datasets (Fig. 2, Extended Data Fig. 2). Other factors, such as cholelithiasis (gallstones) and reflux disease, are perhaps of interest in terms of potential mechanistic hypotheses, such as inflammation of the pancreas before cancer as a result of cholelithiasis or a hypothetical link between medication by proton pump inhibitors, such as omeprazole in reflux disease, and the effect of increased levels of gastrin on the state of the pancreas^43^.

The disease contribution analysis on the US-VA dataset provides a partially overlapping set of top features (Supplementary Table 5b). The disease contribution analysis showed commonly identified disease codes between the two systems, such as unspecified jaundice, other disorders of pancreas, other diseases of biliary tract, diabetes mellitus, abdominal and pelvic pain, other diseases of the liver and symptoms and signs concerning food and fluid intake. Generally, in addition to disease codes that reflect specific risk for pancreatic cancer by current knowledge, others are apparently unspecific, indicating the importance of including many variables with limited individual prognostic value to obtain good aggregate prognostication (Fig. 5b,d).

## Discussion

We present a framework for predicting the risk of a low-incidence but very aggressive cancer by applying deep learning to real-world longitudinal datasets of disease trajectories. This study was designed to make explicit use of the time sequence of disease events and to assess the ability to predict cancer risk for increasing intervals between the end of the disease trajectory used for risk prediction and cancer occurrence. Our results indicate that using the time sequence in disease histories as input to the model, rather than just disease occurrence at any time, improves the ability of AI methods to predict pancreatic cancer occurrence, especially for the highest-risk group (Fig. 3b).

**Extended Data Fig. 5:**
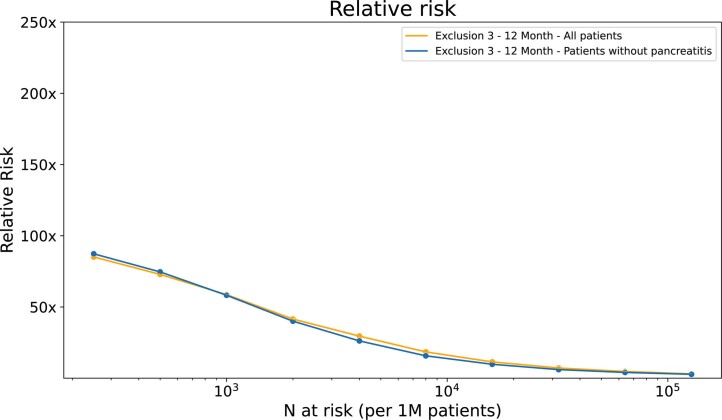
Risk factor for patients without chronic pancreatitis. To assess to what extent the inclusion of people with chronic pancreatitis might boost model performance artificially, we have evaluated model performance for predicting cancer within 12 months for all patients above the age of 50, excluding data from the last three months before the PC diagnosis, for all cases without chronic pancreatitis and closely related conditions (ICD10 codes K86), for comparison with Fig. 4d in the paper. Result: the relative risk remains nearly the same, proving that including patients with chronic pancreatitis does not affect model performance. Moreover, this is supporting evidence for the robustness of the model that bases its prediction not on single diagnoses but rather on the entire set of codes in disease trajectories.

Based on the prediction accuracy reported here, we designed a potentially realistic prediction–surveillance selection process in an example real-world population of 1 million patients with available longitudinal EHRs. The analysis indicates that, by using an ML model trained on all data to predict the 1,000 highest-risk patients (no data exclusion, positive predictive value (PPV) of 0.32; 12-month prediction interval, age 50 years or older), about 320 would eventually develop pancreatic cancer. Some of these might already have been assigned to closer surveillance by their physicians in standard clinical practice based on well-established risk factors, such as chronic pancreatitis (Extended Data Fig. 5). However, a fraction of these would be newly identified as being at high risk—at least about 70 according to a conservative estimate based on the prediction accuracy for models in which the last 3 months of symptoms before cancer occurrence are excluded from input to training (PPV 0.07, 12-month prediction interval, age 50 years or older). Although the precise clinical impact will depend on the quality of EHR data and current clinical practice in a particular healthcare system, we conclude that this level of additional early detection may be considered of value, provided that implementation issues, including the cost of a surveillance program, can be successfully addressed in a real-world implementation (see also the cost estimate in Supplementary Notes).

**Extended Data Fig. 6:**
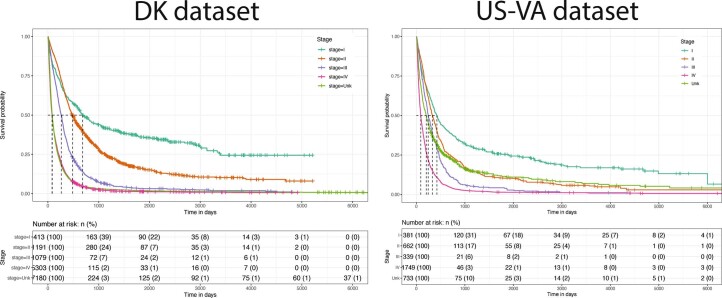
Survival curves. Overall survival in each dataset, stratified by cancer stage. Cases were ascertained using the methods described in Methods, and cancer stage was obtained from the respective dataset’s cancer registry. Stage was only available on a subset of patients.

A single, globally robust model that predicts cancer risk for patients in different countries and different healthcare systems remains elusive. Cross-application of the Danish model to the US-VA database had lower performance in spite of common use of ICD disease codes and similar cancer survival (Fig. 4 and Extended Data Fig. 6). Reasons for this lack of transferability plausibly include differences in clinical practice, such as frequency of reporting disease codes in clinical records, typical thresholds for seeking medical attention, potential influence of billing constraints and billing optimization as well as entry to and departure from the US-VA health system—all of these in contrast to the more uniform and comprehensive nature of the DNPR. However, the AI methods used are sufficiently robust to restore a reasonable level of performance in the US-VA system via independent training. We conclude that, using current methodology, when there are substantial differences in healthcare systems, independent model training in different geographical locations is required to achieve locally optimal model performance. However, if independent training is not feasible—for example, in smaller healthcare systems—then cross-application may be of value, depending on the actual outcome of cross-performance tests, which can be performed on smaller datasets than those required for training.

To achieve a globally useful set of prediction rules, access to large datasets of disease histories aggregated nationally or internationally will be extremely valuable, with careful assessment of the accuracy of clinical records. An ideal scenario for a multi-institutional collaboration would be to employ federated learning across a number of different healthcare systems^44^. Federated learning obviates the need for sharing primary data and only requires permission to run logically identical computer codes at each location and then share and aggregate results. The challenges to achieve federated learning are, however, not only technical but also social and organizational, especially in a competitive healthcare landscape.

Successful implementation of early diagnosis and treatment of pancreatic cancer in clinical practice will likely require three essential steps: (1) identification of high-risk patients; (2) detection of early cancer or pre-cancerous states by detailed surveillance of high-risk patients; and (3) effective treatment after early detection^10,45^. The overall impact in clinical practice depends on the success rates in each of these stages. This work addresses only the first stage. With a reasonably accurate method for predicting cancer risk, one can direct appropriate high-risk patients into surveillance programs. A sufficiently enriched pool of high-risk patients would make detailed screening tests affordable, as such tests are likely to be prohibitively expensive at the population level and enhance the positive yield of such early detection tests.

We expect further increases in prediction accuracy with the real-world availability of data beyond disease codes, such as medication, laboratory values, observations in clinical notes and abdominal imaging (computed tomography and magnetic resonance imaging) as well as population-wide germline genetic profiles and health records from general practitioners^27,46^ and, in the future, patient-provided information about their health state from wearable devices.

The particular advantage of a real-world high-risk prediction–surveillance process is that computational screening of a large population in the first step is inexpensive, whereas the costly second step of sophisticated clinical screening and intervention programs is limited to a much smaller number of patients—those at highest risk. Prediction performance at the level shown here may be sufficient for an initial design of real-world clinical prediction–surveillance programs, and future improvements are likely. AI on real-world clinical records has the potential to produce a scalable workflow for early detection of cancer in the community, to shift focus from treatment of late-stage to early-stage cancer, to improve the quality of life of patients and to increase the benefit/cost ratio of cancer care.

## Methods

### Processing of disease code trajectory datasets

We followed the MI-CLAIM checklist^47^ to improve the reporting of our methods. The checklist is available in Supplementary Table 6.

### Population-level DNPR dataset

The first part of the project was conducted using a dataset of disease histories from the DNPR, covering all 229 million hospital diagnoses of 8.6 million patients between 1977 and 2018. This includes inpatient contacts since 1977 and outpatient and emergency department contacts since 1995 but not data from general practitioners’ records^34^. Each entry of the database includes data on the start and end date of an admission or visit as well as diagnosis codes. The diagnoses are coded according to the ICD (ICD-8 until 1994 and ICD-10 since then). The accuracy of cancer diagnosis disease codes, as examined by the Institute of Clinical Medicine, Aarhus University Hospital, has been reported to be 98% (accuracy for 19 Charlson conditions in 950 reviewed records)^48^. For cancer diagnoses specifically, the reference evaluation was based on detailed comparisons among randomly sampled discharges from five different hospitals and review of a total of 950 samples^34^. We used both the ICD-8 code 157 and ICD-10 code C25, ‘malignant neoplasm of pancreas’, to define pancreatic cancer cases.

### Use of ICD codes

The ICD system has a hierarchical structure, from the most general level—for example, ‘C: Neoplasms’—to the most specific four-character subcategories—for example, ‘C25.1: Malignant neoplasm of body of pancreas’. The Danish version of the ICD-10 is more detailed than the international ICD-10 but less detailed than the clinical modification of the ICD-10 (ICD-10-CM). In this study, we used the three-character category ICD codes (*n* = 2,997) in constructing the predictive models and defined ‘pancreatic cancer patients’ as patients with at least one code under ‘C25: Malignant neoplasm of pancreas’. For the diagnosis codes in the DNPR, we removed disease codes labeled as ‘temporary’ or ‘referral’ (8.3% removed; Extended Data Fig. 1), as these can be misinterpreted when mixed with the main diagnoses and are not valuable for the purposes of this study.

### Filtering the Danish dataset

Danish citizens have, since 1968, been assigned a unique lifetime CPR number, which is useful for linking to person-specific demographic data. Using an encrypted version of this number, we retrieved patient status as to whether patients are active or inactive in the CPR system as well as information related to residence status. We applied a demographic continuity filter. For example, we excluded from consideration residents of Greenland and patients who lack a stable place of residence in Denmark, as these would potentially have discontinuous disease trajectories. By observation time, we mean active use of the healthcare system.

### Subset of Danish dataset used for training

The DNPR dataset comprised a total of 8,110,706 patients, of whom 23,601 had the ICD-10 pancreatic cancer code C25 and 14,720 had the ICD-8 pancreatic cancer code 157. We used both ICD-10 and ICD-8 independently, without semantic mapping, while retaining the pancreatic cancer occurrence label, assuming that ML is able to combine information from both. Subsequently, we removed patients who have too few diagnoses (<5 events). The number of positive patients used for training after applying the length filter are 23,985 (82% ICD-10 and 18% ICD-8) (Fig. 2 and Supplementary Table 1). Coincidentally, this resulted in a more strict filtering for ICD-8 events, which were used only in 1977–1994 in the data. The final dataset was then randomly split into training (80%), development (10%) and test (10%) data, with the condition that all trajectories from a patient were included only in one split group (train/dev/test), to avoid any information leakage between training and development/test datasets.

### Quality control using the Danish Cancer Registry dataset

To perform a quality check of the pancreatic cancer cases in the DNPR, we compared pancreatic cancer cases in the DNPR with pancreatic cancer cases in the Danish Cancer Registry (DCR). The DCR is a population-wide cancer cohort and one of the largest and most comprehensive in the world, which has recorded nationwide cancer incidences from 1943 (refs. ^49,50^). The DCR contains information on diagnosis, cancer type, tumor topography, TNM staging classification and morphology. The purpose of the DCR is to keep track of cancer incidences and mortality in Denmark and to study causes, courses and statistics for treatment improvements in the Danish health system. In 1997, the cancer registry administration was moved from the Danish Cancer Society to the National Board of Health.

We compared the overlap between pancreatic cancer cases in the DNPR versus cases in the DCR to estimate the quality of our case population used for training. Eighty-eight percent of the pancreatic cancer cases used for training overlapped with the cancer registry. Owing to the differing nature of the DCR and DNPR, for which the latter is a more administrative registry with multiple coding purposes, such as hospital reimbursement, monitoring hospital services and patient trajectories and quality assurance, we did not expect these registries to overlap 100%. As there may be correctly labeled cases in the DNPR that did not get captured in the DCR, the 88% concordance is a lower bound on accuracy of the pancreatic cancer ICD-10 codes in the DNPR, which we used as case labels in training.

### Military veterans US-VA dataset

For the US-VA system, we used data from the VA CDW, which collates EHR and cancer registry information on veterans treated at VA facilities nationwide^51^, using methods similar to prior work^52–54^. The CDW includes EHR data from 1999 to the present, originating from the comprehensive range of primary care and specialized services the VA provides at its inpatient and outpatient facilities as well as claims data for care received at outside facilities and paid for by the VA. Each outpatient visit in the database is associated with the date of the visit, and each inpatient visit is associated with an admission and discharge date. Both inpatient and outpatient visits are also associated with ICD codes (ICD-9 before 1 October 2015 and ICD-10 on and after that date) pertaining to the visit.

### US-VA cancer registry and data quality

In addition to EHR data, the CDW also includes cancer registry data. VA cancer registry activities were initiated pursuant to a national directive in 1998, with incident cases annotated retrospectively from 1995 and prospectively until the present^55^. Cases are abstracted manually by trained cancer registrars in accordance with standards of the North American Association of Central Cancer Registries^56^. Potential cases are flagged for review by custom software (OncoTraX), which identifies potential cases automatically based on occurrence of structured data, such as ICD codes, in the EHR. This approach, where semi-automated screening is followed by manual review by trained cancer registrars, results in highly accurate case ascertainment^55,57^. For example, one study found that VA cancer registry data in the CDW had near-perfect accuracy in colorectal cancer case ascertainment by all evaluated measures (PPV, negative predictive value (NPV), sensitivity and specificity) as compared to de novo manual review of 200 potential cases^58^. In contrast, case ascertainment using ICD codes from the EHR had only 58% PPV. Regarding pancreatic cancer case ascertainment specifically, we compared pancreatic cancer cases in the VA cancer registry to cases identified based on ICD codes in the VA EHR. We found that 94.9% of pancreatic cases in the VA cancer registry between 1999 and 2020 had at least one ICD-10 code for pancreatic cancer in the EHR, whereas only 32.4% of patients with a pancreatic cancer ICD code in the EHR were found in the cancer registry. Spurious ICD codes for pancreatic cancer can occur in the EHR due to multiple reasons, including outright error, use of the cancer code for a screening or evaluation visit, use of the cancer code for medical history and use of the cancer code in dual-use scenarios where cancer care is provided outside the VA.

### Filtering the US-VA dataset and subset used for training

VA patients were included in the study as described in the flow chart (Extended Data Fig. 3). Out of all 15,933,326 patients with ≥1 ICD code in the US-VA CDW between 1999 and 2020 and available in our research study database, we randomly sampled a subset of approximately 3 million patients (2,975,110) owing to limitations of the computational resources available. Based on the considerations above, we identified patients with pancreatic cancer as the overlap of those with a diagnosis of pancreatic cancer in the VA cancer registry and those with an ICD code in the VA EHR system. We excluded patients with an ICD code for pancreatic cancer in the VA EHR data who did not have an entry for pancreatic cancer in the VA cancer registry or vice versa, because the status of these patients was unclear. We further excluded patients with short trajectories (<5 events), as in the Danish dataset. This resulted in a final VA dataset of 1,948,209 patients total, including 3,418 patients with pancreatic cancer (Extended Data Fig. 3 and Fig. 2). We randomly allocated patients in the final VA dataset into training (80%), development (10%) and test (10%) sets, with the condition that all trajectories from a patient were included in only one split group (train/dev/test), to avoid any information leakage between training and development/test datasets, as in the Danish dataset.

### Similarity of survival curves in the two healthcare systems

As a check on the quality of pancreatic cancer case ascertainment in the Danish and US-VA datasets, we plotted overall survival in each dataset, stratified by cancer stage (Extended Data Fig. 6). Cancer stage was obtained from the respective dataset’s cancer registry. Stage was available only on a subset of patients. The overall similarity of the survival curves in the two datasets adds confidence to the quality of the data as selected for the comparative study.

### Training

The following processing steps were carried out identically for the DNPR and US-VA datasets. To best exploit the longitudinality of the health/disease records, for each patient the data were augmented by using all continuous partial trajectories of minimal length ≥5 diagnoses from the beginning of their disease history and ending at different timepoints, which we call the time of assessment.

For patients with cancer, we used only trajectories that end before cancer diagnoses. We used a step function annotation indicating cancer occurrence at different timepoints (3, 6, 12, 36 and 60 months) after the end of each partial trajectory. For the positive (‘PC’) cases, this provides the opportunity to learn from disease histories with longer time gaps between the time of assessment and the time of cancer occurrence. For example, for a patient who had pancreatitis a month or two just before the cancer diagnosis, it is of interest to learn which earlier disease codes might have been predictive of cancer occurrence going back at least several months or perhaps years. The latter is also explored by separately retraining of the ML model, excluding data from the last 3 months or 6 months before cancer diagnosis.

For patients without a pancreatic cancer diagnosis, we only include trajectories that end earlier than 2 years before the end of their disease records (due to death or the freeze date of the DNPR data used here). This avoids the uncertainty of cases in which undiagnosed cancer might have existed before the end of the records. The datasets were sampled in small batches for efficient computation, as is customary in ML. Owing to the small number of cases of pancreatic cancer compared to controls, we used balanced sampling from the trajectories of the patients in the training set such that each batch has an approximately equal number of positive (cancer) and negative (non-cancer) trajectories.

### Model development

A desired model for such diagnosis trajectories consists of three parts: embedding of the categorical disease features, encoding time sequence information and assessing the risk of cancer.

We embeded each event denoted with a level-3 ICD code from a partial disease trajectory in a continuous and low-dimensional latent space^59,60^. Such embedding is data driven and trained together with other parts of the model. For ML models not using embedding, each categorical disease was represented in numeric form as a one-hot encoded vector. The longitudinal records of diagnoses allowed us to construct time sequence models with sequential neural networks.

After embedding, each sequence of diagnoses was encoded into a feature vector using different types of sequential layers (gated recurrent units (GRU)), attention layers (Transformer) or simple pooling layers (bag-of-words model, multilayer perceptron model (MLP)). The encoding layer also included age inputs, which have been demonstrated to have a strong association with pancreatic cancer incidence^11^.

Finally, the embedding and encoding layers were connected to a fully connected feedforward (FF) network to make predictions of future cancer occurrence following a given disease history (the bag-of-words model uses only a single linear layer).

The model parameters were trained by minimizing the prediction error quantified as the difference between the observed cancer diagnosis in the form of a step function (0 before the occurrence of cancer and 1 from the time of cancer diagnosis) and the predicted risk score in terms of a positive function that monotonically increases from 0, using a cross-entropy loss function, with the sum over the five timepoints, and L2 regularization on the parameters (Fig. 1a).

$${{{\mathrm{loss}}}}\;\frac{1}{N}\frac{1}{{N_T}}\mathop {\sum}\limits_{i,t} { - \left[ {y_{i,t}\log \left[ {\hat p_{\Theta ,t}(x_i)} \right] + (1 - y_{i,t})\log \left[ {1 - \hat p_{\Theta ,t}(x_i)} \right]} \right] + \lambda _2\left\| \Theta \right\|_2}$$

where $t \in \{ 3,6,12,36,60\}$ is for months; $N_T = 5$ is for non-cancer patients; $N_T \le 5$ is for cancer patients where we use only the timepoints before the cancer diagnosis; $i = 1,2,3,$…,N labels samples; $\Theta$ is the set of model parameters; $\lambda _2$ is the regularization strength; $\hat p$ is the estimated risk output by the model; $x_i$ is the input disease trajectories; $y_{i,t} = 1$ is for cancer occurrence; and $y_{i,t} = 0$ is for no cancer within a *t*-month time window.

The Transformer model, unlike the recurrent models, does not process the input as a sequence of timesteps but, rather, uses an attention mechanism to enhance the embedding vectors correlated with the outcome. To enable the Transformer to digest temporal information, such as the order of the exact dates of the diseases inside the sequence, we used positional embedding to encode the temporal information into vectors, which were then used as weights for each disease token. Here, we adapted the positional embedding from ref. ^38^ using the values taken by cosine waveforms at 128 frequencies observed at different times. The times used to extract the wave values were the age at which each diagnosis was administered and the time difference between each diagnosis. In this way, the model is enabled to distinguish between the same disease assigned at different times and two different disease diagnoses far and close in time. The parameters in the embedding machine, which address the issue of data representation suitable for input into a deep learning network, were trained together with the encoding and prediction parts of the model with back-propagation (Fig. 2).

To comprehensively test different types of neural networks and the corresponding hyperparameters, we conducted a large parameter search for each of the network types (Supplementary Table 2). The different types of models include simple FF models (linear regression (LR) and MLP) and more complex models that can take the sequential information of disease ordering into consideration (GRU and Transformer). See the supplementary table with comparison metrics across different models (Supplementary Table 3). To estimate the uncertainty of the performances, the 95% confidence interval was constructed using 200 resamples of bootstrapping with replacement.

### Evaluation

The evaluation was carried out separately for each prediction interval of 0–3, 0–6, 0–12, 0–36 and 0–60 months. For example, consider the prediction score for a particular trajectory at the end of the 3-year prediction interval (Fig. 1c). If the score is above the threshold, one has a correct positive prediction if cancer has occurred at any time within 3 years and a false-positive prediction if cancer has not occurred within 3 years. If the score is below the threshold, one has a false-negative prediction if cancer has occurred at any time within 3 years and a true-negative prediction if cancer has not occurred within 3 years. As both training and evaluation make use of multiple trajectories per patient, with different end-of-trajectory points, the performance numbers were computed over trajectories. For each hyperparameter search, the best model was selected using the area under the precision recall curve (AUPRC).

The relative risk ratio (RR) is calculated as the odds of getting pancreatic cancer when classified at high risk compared to a random method that just uses the disease incidence in the population. RR is defined as

$$RR = \frac{{precision}}{{incidence}} = \frac{{TP/(TP + FP)}}{{(TP + FN)/(TP + FP + TN + FN)}}$$

where TP is true positives, FP is false positives, FN is false negatives and TN is true negatives. The RR score is defined at a given operational decision point along the RR curve as a function of the number of patients predicted to be at high risk (Fig. 3, right, and Fig. 4). The RR ratio quantifies by what factor a prediction method does better than a random pick based just on the population disease incidence.

For the purpose of comparing different algorithms, input modes and DNPR versus US-VA, we report exhaustive performance tables (Supplementary Table 3a–i) with precision and recall at the F1 operational point, which maximizes the harmonic mean of recall and precision^61^. However, for consideration of clinical implementation, which requires severe limits on the number of patients who can be advanced to a surveillance program, we use an operational point for the top 1,000 high-risk patients out of 1 million patients (0.1% of 1 million; see RR plots: Figs. 3 and 4).

### Cross-application to the US-VA dataset

To assess the predictive performance of the model in other healthcare systems, we applied the best ML model trained on the DNPR to disease trajectories of patients in the US-VA dataset. For the US-VA dataset, we directly applied models without any adaptation except for mapping the ICD code from one system to the other. In brief, ICD-9 codes in the US-VA dataset were first mapped to ICD-10 codes, followed by adding a prefix ‘D’ to obtain Denmark-compatible ICD-10 codes.

### Interpreting clinically relevant features

To find the features that are strongly associated with pancreatic cancer, we used an attribution method for neural networks called integrated gradients^40^. This method calculates the contribution of input features, called attribution, cumulating the gradients calculated along all the points in the path from the input to the baseline. We chose the output of interest to be the 36-month prediction. Positive and negative attribution scores (contribution to prediction) indicate positive and negative correlation with patients with pancreatic cancer. Because the gradient cannot be calculated with respect to the indices used as input of the embedding layer, the input used for the attribution was the output of the embedding layer. Then, the attribution at the token level was obtained summing up over each embedding dimension and summing across all the patient trajectories. Similarly, for each trajectory, we calculated the age contribution as the sum attribution of the integrated gradients of the input at the age embedding layer.

### Reporting summary

Further information on research design is available in the Nature Portfolio Reporting Summary linked to this article.

## Online content

Any methods, additional references, Nature Portfolio reporting summaries, source data, extended data, supplementary information, acknowledgements, peer review information; details of author contributions and competing interests; and statements of data and code availability are available at 10.1038/s41591-023-02332-5.

## Supplementary information

Supplementary Information Supplementary Tables 1–6 and Supplementary Notes

Reporting Summary

## Data Availability

Danish registry-based studies do not require ethical approvals, and informed consent is not required. This study has been approved by the Danish Health Data Authority (FSEID-00003092 and FSEID-00004491), the Danish Data Protection Agency (ref: SUND-2017-57) and General Data Protection Regulation record of processing activity (ref: 514-0255/18-3000). Application for data access can be made to the Danish Health Data Authority (contact: servicedesk@sundhedsdata.dk). Anyone wanting access to the data and to use them for research will be required to meet research credentialing requirements as outlined at the authority’s web site: https://sundhedsdatastyrelsen.dk/da/english/health_data_and_registers/research_services. Requests are normally processed within 3–6 months. Analysis in the US-VA system was conducted under a waiver of informed consent with approval from the VA Boston Healthcare System Institutional Review Board. All VA data used in this study are available to any investigator upon relevant approvals through the VA Informatics and Computing Infrastructure (VINCI) (contact: VINCI@va.gov). Anyone wanting access to the data and to use them for research will be required to meet research credentialing requirements as outlined by the VA Office of Research and Development, which is expected to be processed within at least 6 months.

## Code availability

The software, under the name CancerRiskNet (initial commit 22 February 2023), is freely available at https://github.com/BrunakSanderLabs/CancerRiskNet. All analyses carried out in this study were performed using Python version 3.8.10. The Python package torch version l.9.0 was used for developing the machine learning models, and integrated gradient was implemented using the Python package Captum version 0.3.1. Visualization was obtained using Python packages matplotlib version 3.3.2 and seaborn version 0.11.0.

## References

[1] Rahib L (2014). Projecting cancer incidence and deaths to 2030: the unexpected burden of thyroid, liver, and pancreas cancers in the United States. Cancer Res..

[2] McGuigan A (2018). Pancreatic cancer: a review of clinical diagnosis, epidemiology, treatment and outcomes. World J. Gastroenterol..

[3] Amundadottir L (2009). Genome-wide association study identifies variants in the *ABO* locus associated with susceptibility to pancreatic cancer. Nat. Genet..

[4] Petersen GM (2010). A genome-wide association study identifies pancreatic cancer susceptibility loci on chromosomes 13q22.1, 1q32.1 and 5p15.33. Nat. Genet..

[5] Li D (2012). Pathway analysis of genome-wide association study data highlights pancreatic development genes as susceptibility factors for pancreatic cancer. Carcinogenesis.

[6] Wolpin BM (2014). Genome-wide association study identifies multiple susceptibility loci for pancreatic cancer. Nat. Genet..

[7] Klein AP (2018). Genome-wide meta-analysis identifies five new susceptibility loci for pancreatic cancer. Nat. Commun..

[8] Kim J (2020). Genetic and circulating biomarker data improve risk prediction for pancreatic cancer in the general population. Cancer Epidemiol. Biomark. Prev..

[9] Pereira SP (2020). Early detection of pancreatic cancer. Lancet Gastroenterol. Hepatol..

[10] Singhi AD, Koay EJ, Chari ST, Maitra A (2019). Early detection of pancreatic cancer: opportunities and challenges. Gastroenterology.

[11] Klein, A. P. Pancreatic cancer epidemiology: understanding the role of lifestyle and inherited risk factors. *Nat. Rev. Gastroenterol. Hepatol*. **18**, 493–502 (2021). 10.1038/s41575-021-00457-x PMC9265847 34002083

[12] Chen F, Roberts NJ, Klein AP (2017). Inherited pancreatic cancer. Chin. Clin. Oncol..

[13] Dietterich, T. G. Machine learning for sequential data: a review. In *Structural, Syntactic, and Statistical Pattern Recognition* (eds Caelli, T., Amin, A., Duin, R. P. W., Ridder, D. & Kamel, M.) 15–30 (Springer, 2002).

[14] LeCun Y, Bengio Y, Hinton G (2015). Deep learning. Nature.

[15] Nielsen AB (2019). Survival prediction in intensive-care units based on aggregation of long-term disease history and acute physiology: a retrospective study of the Danish National Patient Registry and electronic patient records. Lancet Digit. Health.

[16] Thorsen-Meyer H-C (2020). Dynamic and explainable machine learning prediction of mortality in patients in the intensive care unit: a retrospective study of high-frequency data in electronic patient records. Lancet Digit. Health.

[17] Shickel B, Tighe PJ, Bihorac A, Rashidi P (2018). Deep EHR: a survey of recent advances in deep learning techniques for electronic health record (EHR) analysis. IEEE J. Biomed. Health Inform..

[18] Hyland SL (2020). Early prediction of circulatory failure in the intensive care unit using machine learning. Nat. Med..

[19] Esteva A (2017). Dermatologist-level classification of skin cancer with deep neural networks. Nature.

[20] Yala A, Lehman C, Schuster T, Portnoi T, Barzilay R (2019). A deep learning mammography-based model for improved breast cancer risk prediction. Radiology.

[21] Yamada M (2019). Development of a real-time endoscopic image diagnosis support system using deep learning technology in colonoscopy. Sci. Rep..

[22] Jung, A. W. et al. Multi-cancer risk stratification based on national health data: a retrospective modelling and validation study. Preprint at *bioRxiv*10.1101/2022.10.12.22280908 (2022). 10.1016/S2589-7500(24)00062-1 38789140

[23] Tomašev N (2019). A clinically applicable approach to continuous prediction of future acute kidney injury. Nature.

[24] Li Y (2020). BEHRT: transformer for electronic health records. Sci. Rep..

[25] Thorsen-Meyer H-C (2022). Discrete-time survival analysis in the critically ill: a deep learning approach using heterogeneous data. NPJ Digit. Med..

[26] Muhammad W (2019). Pancreatic cancer prediction through an artificial neural network. Front. Artif. Intell..

[27] Malhotra A, Rachet B, Bonaventure A, Pereira SP, Woods LM (2021). Can we screen for pancreatic cancer? Identifying a sub-population of patients at high risk of subsequent diagnosis using machine learning techniques applied to primary care data. PLoS ONE.

[28] Appelbaum L (2021). Development and validation of a pancreatic cancer risk model for the general population using electronic health records: an observational study. Eur. J. Cancer.

[29] Li X (2020). A deep-learning based prediction of pancreatic adenocarcinoma with electronic health records from the state of Maine. Int. J. Med. Health Sci..

[30] Chen Q (2021). Clinical data prediction model to identify patients with early-stage pancreatic cancer. JCO Clin. Cancer Inform..

[31] Appelbaum, L. et al. Development of a pancreatic cancer prediction model using a multinational medical records database. *J. Clin. Oncol.*10.1200/JCO.2021.39.3_suppl.394 (2021).

[32] Hu JX, Helleberg M, Jensen AB, Brunak S, Lundgren J (2019). A large-cohort, longitudinal study determines precancer disease routes across different cancer types. Cancer Res..

[33] Jensen AB (2014). Temporal disease trajectories condensed from population-wide registry data covering 6.2 million patients. Nat. Commun..

[34] Schmidt M (2015). The Danish National Patient Registry: a review of content, data quality, and research potential. Clin. Epidemiol..

[35] Siggaard T (2020). Disease trajectory browser for exploring temporal, population-wide disease progression patterns in 7.2 million Danish patients. Nat. Commun..

[36] Schmidt M, Pedersen L, Sørensen HT (2014). The Danish Civil Registration System as a tool in epidemiology. Eur. J. Epidemiol..

[37] Cho, K. et al. Learning phrase representations using RNN encoder–decoder for statistical machine translation. Preprint at *arXiv*10.48550/arXiv.1406.1078 (2014).

[38] Vaswani, A. et al. Attention is all you need. *31st Conference on Neural Information Processing Systems* (NIPS, 2017).

[39] Yuan C (2020). Diabetes, weight change, and pancreatic cancer risk. JAMA Oncol..

[40] Sundararajan, M., Taly, A. & Yan, Q. Axiomatic attribution for deep networks. *Proc. 34th Intl. Conf. Mach.Learning* (JMLR, 2017).

[41] Klein AP (2013). An absolute risk model to identify individuals at elevated risk for pancreatic cancer in the general population. PLoS ONE.

[42] Hjaltelin, J. X. et al. Pancreatic cancer symptom trajectories from Danish registry data and free text in electronic health records. Preprint at *medRxiv*10.1101/2023.02.13.23285861 (2023). 10.7554/eLife.84919 PMC10662947 37988407

[43] Alkhushaym N (2020). Exposure to proton pump inhibitors and risk of pancreatic cancer: a meta-analysis. Expert Opin. Drug Saf..

[44] Konečný, J. et al. Federated learning: strategies for improving communication efficiency. Preprint at *arXiv*10.48550/arXiv.1610.05492 (2016).

[45] Kenner B (2021). Artificial intelligence and early detection of pancreatic cancer: 2020 summative review. Pancreas.

[46] Lemanska A (2022). BMI and HbA1c are metabolic markers for pancreatic cancer: matched case–control study using a UK primary care database. PLoS ONE.

[47] Norgeot B (2020). Minimum information about clinical artificial intelligence modeling: the MI-CLAIM checklist. Nat. Med..

[48] Thygesen SK, Christiansen CF, Christensen S, Lash TL, Sørensen HT (2011). The predictive value of ICD-10 diagnostic coding used to assess Charlson comorbidity index conditions in the population-based Danish National Registry of Patients. BMC Med. Res. Methodol..

[49] Gjerstorff ML (2011). The Danish Cancer Registry. Scand. J. Public Health.

[50] Sundhedsstyrelsen. Det moderniserede Cancerregister—metode og kvalitet. https://sundhedsdatastyrelsen.dk/-/media/sds/filer/registre-og-services/nationale-sundhedsregistre/sygdomme-laegemidler-og-behandlinger/cancerregisteret/det-moderniserede-cancerregister.pdf?la=da#:~:text=Et%20af%20de%20overordnede%20form%C3%A5l,%2C%20komplethed%2C%20rettidighed%20og%20sammenlignelighed. (2009).

[51] Price LE, Shea K, Gephart S (2015). The Veterans Affairs’s Corporate Data Warehouse: uses and implications for nursing research and practice. Nurs. Adm. Q..

[52] Elbers DC (2020). The Veterans Affairs Precision Oncology Data Repository, a clinical, genomic, and imaging research database. Patterns (N Y).

[53] Chang MS (2022). Increased relative proportions of advanced melanoma among veterans: a comparative analysis with the Surveillance, Epidemiology, and End Results registry. J. Am. Acad. Dermatol..

[54] Wu JT-Y (2022). Association of COVID-19 vaccination with SARS-CoV-2 infection in patients with cancer: a US nationwide Veterans Affairs study. JAMA Oncol..

[55] Zullig LL (2012). Cancer incidence among patients of the U.S. Veterans Affairs Health Care System. Mil. Med..

[56] *Standards for Cancer Registries Volume II: Data Standards and Data Dictionary*. 24th edn, Ver. 23 (ed Thornton, M.) https://datadictionary.naaccr.org/default.aspx?c=1&Version=23 (North American Association of Central Cancer Registries, 2022).

[57] Zullig LL (2019). Summary of Veterans Health Administration cancer data sources. J. Registry Manag..

[58] Earles A (2018). Structured approach for evaluating strategies for cancer ascertainment using large-scale electronic health record data. JCO Clin. Cancer Inform..

[59] Mikolov, T., Chen, K., Corrado, G. & Dean, J. Efficient estimation of word representations in vector space. Preprint at *arXiv*10.48550/arXiv.1301.3781 (2013).

[60] Gehring, J., Auli, M., Grangier, D., Yarats, D. & Dauphin, Y. N. Convolutional sequence to sequence learning. In *Proc. of the 34th International Conference on Machine Learning* (eds Precup, D. & Teh, Y. W.) 1243–1252 (PMLR, 2017).

[61] Sasaki, Y (The truth of the F-measure. https://www.cs.odu.edu/~mukka/cs795sum11dm/Lecturenotes/Day3/F-measure-YS-26Oct07.pdf (School of Computer Science, Univ. of Manchester: 2007.

