# Supplementary material for: A deep learning algorithm to predict risk of pancreatic cancer from disease trajectories
Source: Nat Med. 2023 May 8;29(5):1113–22. doi: 10.1038/s41591-023-02332-5 (PMC10202814; doi:10.1038/s41591-023-02332-5)
Supplement: Supplementary file 1 — Supplementary Tables 1–6 and Supplementary Notes [file 41591_2023_2332_MOESM1_ESM.pdf]

nature medicine

Article https://doi.org/10.1038/s41591-023-02332-5

# A deep learning algorithm to predict risk of pancreatic cancer from disease trajectories

In the format provided by the authors and unedited

# Supplementary Tables and Notes

## Supplementary tables

### Supplementary table 1. Description of the patient cohorts used in this study (DK).

| Population Metadata (n=8,110,706 persons) | | |
|---|---|---|
| Gender | Male | Female |
| Total Count | 4,030,504 (49.69%) | 4,080,202 (50.31%) |
| Alive | 2,754,152 (33.96%) | 2,827,021 (34.86% ) |
| Dead | 1,276,352 (15.74%) | 1,253,181 (15.45%) |
| After continuity and length filtering | 2,938,248 (36.23%) | 3,239,989 (39.95%) |
| Age at last record [0-10) | 216,329 (2.67%) | 204,774 (2.52%) |
| Age at last record [10-20) | 332,326 (4.10%) | 314,445 (3.88%) |
| Age at last record [20-30) | 322,802 (3.98%) | 298,219 (3.68%) |
| Age at last record [30-40) | 283,200 (3.49%) | 305,470 (3.77%) |
| Age at last record [40-50) | 323,811 (3.99%) | 380,730 (4.69%) |
| Age at last record [50-60) | 368,686 (4.55%) | 419,100 (5.17%) |
| Age at last record [60-70) | 373,220 (4.60%) | 402,625 (4.96%) |
| Age at last record [70-80) | 394,789 (4.87%) | 408,890 (5.04%) |
| Age at last record [80-90) | 258,193 (3.18%) | 342,174 (4.22%) |
| Age at last record [90-100) | 63,470 (0.78%) | 156,154 (1.93%) |
| Age at last record [100, ∞) | 1,422 (0.02%) | 7,398 (0.09%) |

| Pancreatic Cancer Patients (n=23,985) | | |
|---|---|---|
| | Male | Female |
| Total Count | 11,880 (49.53%) | 12,105 50.47% |
| Age at pancreatic cancer diagnosis [0,30) | 13 (0.05%) | 19 (0.08%) |
| Age at pancreatic cancer diagnosis [30-40) | 92 (0.38%) | 93 (0.39%) |
| Age at pancreatic cancer diagnosis [40-50) | 474 (1.98%) | 417 (1.74%) |
| Age at pancreatic cancer diagnosis [50-60) | 1,626 (6.78%) | 1,304 (5.44%) |
| Age at pancreatic cancer diagnosis [60-70) | 3,585 (14.95%) | 2,950 (12.30%) |
| Age at pancreatic cancer diagnosis [70-80) | 4,017 (16.75%) | 4,076 (16.99%) |
| Age at pancreatic cancer diagnosis [80-90) | 1,925 (8.03%) | 2,751 (11.47%) |
| Age at pancreatic cancer diagnosis [90, ∞) | 148 (0.62%) | 495 (2.06%) |

**Supplementary table 2. Hyperparameter search for machine learning models.**

To comprehensively test different types of neural networks and the corresponding hyperparameters, we conducted a large parameter search for each of the network types. The different types of models include simple feed-forward models (LR, MLP) and more complex models that can take the sequential information of disease ordering into consideration (RNN, GRU and Transformer). The hyperparameters of the best performing model (DK-DNPR) are in **bold**.

| | **Type of ML model** | | | |
|---|---|---|---|---|
| **Hyper-parameters** | Bag of words | MLP | GRU | Transformer |
| Dropout | 0 | 0,0.1 | 0,0.1 | **0**, 0.1 |
| Weight decay | 0.001 | 0,0.001 | 0,0.001 | 0, **0.001** |
| Only prior knowledge diseases | False, True | False | False | **False**, True |
| Dimension of hidden layer | - | 32, 128, 256 | 32, 64, 128, 256 | 32, **256** |
| Number of hidden layers | - | 1, 2 | 1, 2, 4 | **1**, 2, 4 |
| Age input | None | None | None, positional embedding | None, **positional embedding** |
| Time input | None | None | None, positional embedding | None, **positional embedding** |
| Number of Heads | - | - | - | 8, **16**, 32 |

## Supplementary table 3. Performance of exclusion experiments - DK dataset and US-VA dataset

A summary of performance of different models trained with different data exclusion intervals for different prediction intervals. In order to estimate the uncertainty of the performance metrics, 95% confidence interval (CI) were computed using 200 resamples (bootstrapping with replacement); these time intervals may be slightly too narrow due to the estimated small number of trajectories from a single patient in a particular sample, but provide a reasonable guide. Specificity, precision, and recall are for the F1-optimal operational point that maximizes the F1 score, which is the harmonic mean of recall and precision [1].

Summary of Tables S3

| | |
|---|---|
| Table S3A | Performance summary DNPR (AUROC) |
| Table S3B | Performance summary DNPR (Specificity/Precision/Recall) |
| Table S3C | Performance summary DNPR models applied to US-VA (AUROC) |
| Table S3D | Performance summary DNPR models applied to US-VA (Specificity/Precision/Recall) |
| Table S3E | Performance summary US-VA, independent training (AUROC) |
| Table S3F | Performance summary US-VA, independent training (Specificity/Precision/Recall) |
| Table S3G | Performance summary US-VA, independent training above 50 (AUROC) |
| Table S3H | Performance summary US-VA, independent training above 50 (Specificity/Precision/Recall) |
| Table S3I | Performance summary DNPR above 50 (AUROC) |
| Table S3J | Performance summary DNPR above 50 (Specificity/Precision/Recall) |

## Table S3A Performance summary DNPR (AUROC)

| Model | Prediction Interval (months) → | 0 - 3 | 0 - 6 | 0 - 12 | 0 - 36 | 0 - 60 |
|---|---|---|---|---|---|---|
| | Exclusion Interval (months) | | | | | |
| **Bag-of-words** | 0 | 0.794 (0.791-0.797) | 0.800 (0.797-0.803) | 0.807 (0.805-0.809) | 0.807 (0.805-0.809) | 0.799 (0.797-0.800) |
| | 3 | - | 0.815 (0.808-0.821) | 0.823 (0.819-0.826) | 0.812 (0.810-0.814) | 0.798 (0.796-0.800) |
| | 6 | - | - | 0.826 (0.821-0.830) | 0.810 (0.807-0.812) | 0.794 (0.792-0.797) |
| **MLP** | 0 | 0.876 (0.873-0.879) | 0.871 (0.869-0.873) | 0.864 (0.861-0.866) | 0.845 (0.843-0.847) | 0.832 (0.830-0.834) |
| | 3 | - | 0.836 (0.830-0.841) | 0.832 (0.828-0.836) | 0.838 (0.836-0.840) | 0.828 (0.827-0.830) |
| | 6 | - | - | 0.838 (0.833-0.842) | 0.830 (0.828-0.833) | 0.824 (0.822-0.825) |
| **GRU** | 0 | 0.917 (0.914-0.919) | 0.903 (0.900-0.905) | 0.883 (0.881-0.885) | 0.852 (0.850-0.854) | 0.836 (0.834-0.837) |
| | 3 | - | 0.859 (0.854-0.866) | 0.852 (0.848-0.855) | 0.832 (0.830-0.835) | 0.820 (0.818-0.822) |
| | 6 | - | - | 0.848 (0.844-0.852) | 0.827 (0.824-0.829) | 0.815 (0.812-0.816) |
| | 12 | - | - | - | 0.814 (0.811-0.817) | 0.803 (0.801-0.805) |
| **Transformer** | 0 | 0.934 (0.932-0.937) | 0.923 (0.920-0.925) | 0.908 (0.906-0.911) | 0.879 (0.877-0.880) | 0.861 (0.860-0.863) |
| | 3 | - | 0.866 (0.860-0.870) | 0.862 (0.857-0.866) | 0.843 (0.841-0.844) | 0.830 (0.828-0.831) |
| | 6 | - | - | 0.834 (0.830-0.838) | 0.829 (0.827-0.832) | 0.817 (0.816-0.819) |
| | 12 | - | - | - | 0.827 (0.825-0.830) | 0.816 (0.814-0.818) |
| **Transformer - Known risk factors** | 0 | 0.850 (0.847-0.852) | 0.850 (0.847-0.852) | 0.850 (0.848-0.851) | 0.838 (0.837-0.840) | 0.832 (0.831-0.833) |

## Table S3B Performance summary DNPR (Specificity/Precision/Recall)

| Model | | Prediction Interval (months) : → | 0 - 3 | 0 - 6 | 0 - 12 | 0 - 36 | 0 - 60 |
|---|---|---|---|---|---|---|---|
| | Exclusion Interval (months) | Metric | | | | | |
| **Bag-of-words** | 0 | specificity | 98.64% (96.31%-98.83%) | 98.07% (95.42%-98.85%) | 98.18% (97.50%-98.80%) | 95.55% (94.86%-98.01%) | 95.06% (94.09%-95.75%) |
| | 0 | precision | 0.3% (0.3%-0.4%) | 0.4% (0.4%-0.5%) | 0.6% (0.6%-0.7%) | 0.9% (0.8%-0.9%) | 1.0% (0.9%-1.0%) |
| | 0 | recall | 5.4% (4.6%-13.4%) | 8.0% (4.9%-17.5%) | 7.7% (5.3%-9.9%) | 16.6% (8.1%-18.6%) | 16.5% (14.6%-19.2%) |
| | 3 | specificity | - | 99.91% (99.80%-99.91%) | 99.72% (99.15%-99.80%) | 97.04% (94.91%-99.70%) | 94.82% (93.27%-97.03%) |
| | 3 | precision | - | 0.2% (0.1%-0.3%) | 0.4% (0.3%-0.5%) | 0.6% (0.6%-0.9%) | 0.7% (0.7%-0.7%) |
| | 3 | recall | - | 1.0% (0.7%-2.1%) | 2.0% (1.4%-4.9%) | 11.7% (1.8%-19.4%) | 17.2% (10.2%-22.2%) |
| | 6 | specificity | - | - | 99.73% (99.19%-99.74%) | 99.71% (97.11%-99.72%) | 96.72% (93.37%-97.43%) |
| | 6 | precision | - | - | 0.2% (0.2%-0.3%) | 0.7% (0.5%-0.8%) | 0.6% (0.6%-0.7%) |
| | 6 | recall | - | - | 2.1% (1.7%-5.2%) | 1.7% (1.6%-11.6%) | 10.8% (8.5%-20.7%) |
| **MLP** | 0 | specificity | 99.74% (99.68%-99.82%) | 99.73% (99.66%-99.82%) | 99.79% (99.66%-99.82%) | 99.69% (99.53%-99.74%) | 99.54% (99.43%-99.61%) |
| | 0 | precision | 2.7% (2.4%-3.0%) | 3.4% (3.0%-3.9%) | 4.3% (3.6%-4.7%) | 4.8% (4.1%-5.3%) | 4.5% (4.2%-4.9%) |
| | 0 | recall | 9.0% (6.9%-11.1%) | 9.1% (7.0%-11.1%) | 7.3% (6.6%-9.8%) | 7.3% (6.5%-9.4%) | 7.9% (7.1%-9.0%) |
| | 3 | specificity | - | 99.85% (99.72%-99.87%) | 99.83% (99.71%-99.84%) | 99.75% (99.50%-99.76%) | 99.43% (99.39%-99.56%) |
| | 3 | precision | - | 0.5% (0.4%-0.6%) | 1.0% (0.9%-1.2%) | 2.0% (1.5%-2.1%) | 1.8% (1.7%-2.0%) |
| | 3 | recall | - | 3.5% (2.8%-5.4%) | 3.3% (2.9%-4.8%) | 3.7% (3.4%-5.9%) | 5.1% (4.3%-5.6%) |
| | 6 | specificity | - | - | 99.80% (99.80%-99.82%) | 99.64% (99.62%-99.92%) | 99.61% (99.59%-99.64%) |
| | 6 | precision | - | - | 0.3% (0.3%-0.4%) | 1.0% (0.9%-1.9%) | 1.3% (1.2%-1.4%) |
| | 6 | recall | - | - | 2.0% (1.5%-2.4%) | 3.1% (1.3%-3.5%) | 2.8% (2.6%-3.1%) |
| **GRU** | 0 | specificity | 99.95% (99.93%-99.95%) | 99.92% (99.89%-99.94%) | 99.89% (99.87%-99.91%) | 99.82% (99.77%-99.87%) | 99.76% (99.74%-99.81%) |
| | 0 | precision | 15.1% (13.1%-15.9%) | 14.0% (11.7%-15.9%) | 13.1% (12.0%-14.6%) | 11.6% (10.2%-13.5%) | 10.4% (9.8%-11.5%) |
| | 0 | recall | 12.7% (11.9%-14.0%) | 12.6% (11.4%-14.7%) | 12.6% (11.4%-13.5%) | 10.8% (9.5%-12.0%) | 10.0% (9.1%-10.5%) |
| | 3 | specificity | - | 99.97% (99.93%-99.97%) | 99.94% (99.91%-99.95%) | 99.86% (99.83%-99.89%) | 99.84% (99.79%-99.86%) |
| | 3 | precision | - | 2.8% (2.2%-3.4%) | 5.2% (4.1%-6.0%) | 5.5% (4.9%-6.2%) | 5.8% (5.0%-6.3%) |
| | 3 | recall | - | 4.9% (4.2%-7.1%) | 6.1% (5.3%-7.6%) | 6.0% (5.1%-6.7%) | 5.1% (4.7%-5.7%) |
| | 6 | specificity | - | - | 99.93% (99.85%-99.96%) | 99.88% (99.85%-99.93%) | 99.84% (99.78%-99.85%) |
| | 6 | precision | - | - | 1.7% (1.3%-2.2%) | 4.3% (3.5%-5.5%) | 4.3% (3.7%-4.7%) |
| | 6 | recall | - | - | 3.8% (2.8%-5.6%) | 4.4% (3.5%-5.3%) | 4.2% (3.9%-4.8%) |
| | 12 | specificity | - | - | - | 99.67% (99.58%-99.89%) | 99.79% (99.47%-99.84%) |
| | 12 | precision | - | - | - | 1.1% (0.9%-1.3%) | 1.7% (1.2%-1.9%) |
| | 12 | recall | - | - | - | 4.4% (2.0%-5.1%) | 2.6% (2.2%-4.6%) |
| **Transformer** | 0 | specificity | 99.95% (99.92%-99.96%) | 99.93% (99.91%-99.94%) | 99.92% (99.90%-99.93%) | 99.88% (99.87%-99.90%) | 99.87% (99.83%-99.88%) |
| | 0 | precision | 18.6% (15.6%-22.5%) | 18.8% (16.9%-19.7%) | 19.4% (17.8%-21.6%) | 18.1% (17.1%-19.9%) | 18.0% (15.2%-18.9%) |
| | 0 | recall | 16.5% (14.4%-19.4%) | 17.0% (16.1%-18.5%) | 15.6% (14.7%-16.5%) | 12.3% (11.7%-12.9%) | 10.2% (9.8%-11.2%) |
| | 3 | specificity | - | 99.92% (99.91%-99.98%) | 99.92% (99.92%-99.93%) | 99.87% (99.86%-99.91%) | 99.63% (99.56%-99.64%) |
| | 3 | precision | - | 1.7% (1.4%-3.0%) | 4.3% (3.8%-4.8%) | 5.4% (4.9%-6.6%) | 2.7% (2.5%-2.9%) |
| | 3 | recall | - | 5.9% (2.4%-7.2%) | 6.5% (6.0%-7.2%) | 5.2% (4.5%-5.6%) | 5.3% (5.0%-6.0%) |
| | 6 | specificity | - | - | 99.41% (98.21%-99.42%) | 99.51% (99.47%-99.52%) | 99.34% (95.82%-99.38%) |
| | 6 | precision | - | - | 0.2% (0.1%-0.2%) | 0.7% (0.7%-0.8%) | 0.8% (0.7%-0.9%) |
| | 6 | recall | - | - | 3.4% (2.7%-8.4%) | 3.2% (2.9%-3.5%) | 3.2% (3.0%-16.0%) |
| | 12 | specificity | - | - | - | 99.44% (99.43%-99.45%) | 99.41% (94.87%-99.42%) |
| | 12 | precision | - | - | - | 0.5% (0.4%-0.5%) | 0.6% (0.5%-0.7%) |
| | 12 | recall | - | - | - | 3.1% (2.8%-3.5%) | 2.7% (2.5%-18.3%) |
| **Transformer - Known risk factors** | 0 | specificity | 99.96% (99.92%-99.97%) | 99.92% (99.91%-99.93%) | 99.91% (99.91%-99.92%) | 99.87% (99.76%-99.88%) | 99.79% (99.73%-99.88%) |
| | 0 | precision | 11.6% (7.4%-12.7%) | 9.2% (8.6%-10.0%) | 10.3% (9.7%-10.8%) | 3.6% (2.6%-3.9%) | 2.8% (2.4%-4.0%) |
| | 0 | recall | 6.9% (6.3%-9.7%) | 9.2% (8.5%-9.7%) | 8.3% (7.9%-8.7%) | 2.5% (2.3%-3.2%) | 2.4% (1.9%-2.8%) |

## Table S3C Performance summary DNPR models applied to US-VA (AUROC)

| Model | Prediction Interval (months) → Exclusion Interval (months) | 0 - 3 | 0 - 6 | 0 - 12 | 0 - 36 | 0 - 60 |
|---|---|---|---|---|---|---|
| Transformer | 0 | 0.881 (0.878-0.883) | 0.833 (0.830-0.835) | 0.790 (0.788-0.792) | 0.710 (0.708-0.712) | 0.685 (0.683-0.687) |
| | 3 | - | 0.665 (0.661-0.670) | 0.641 (0.638-0.643) | 0.626 (0.624-0.628) | 0.627 (0.625-0.629) |
| | 6 | - | - | 0.593 (0.590-0.598) | 0.615 (0.612-0.616) | 0.609 (0.607-0.610) |

## Table S3D Performance summary DNPR models applied to US-VA (Specificity/Precision/Recall)

| Model | Exclusion Interval (months) | Prediction Interval (months): → Metric | 0 - 3 | 0 - 6 | 0 - 12 | 0 - 36 | 0 - 60 |
|---|---|---|---|---|---|---|---|
| Transformer (cross evaluation) | 0 | specificity | 99.94% (99.92%-99.97%) | 99.92% (99.91%-99.92%) | 99.91% (99.91%-99.92%) | 99.90% (99.90%-99.91%) | 99.90% (99.90%-99.90%) |
| | 0 | precision | 6.8% (5.9%-9.3%) | 6.1% (5.8%-6.4%) | 6.4% (6.2%-6.7%) | 6.2% (6.0%-6.4%) | 6.3% (6.1%-6.4%) |
| | 0 | recall | 13.3% (9.1%-17.7%) | 12.9% (12.4%-13.4%) | 9.7% (9.2%-10.0%) | 5.9% (5.7%-6.1%) | 4.9% (4.7%-5.0%) |
| | 3 | specificity | - | 99.90% (99.90%-99.90%) | 99.90% (99.90%-99.90%) | 99.90% (99.90%-99.90%) | 99.86% (99.72%-99.86%) |
| | 3 | precision | - | 0.4% (0.4%-0.5%) | 0.8% (0.7%-0.9%) | 1.3% (1.2%-1.4%) | 1.4% (1.1%-1.5%) |
| | 3 | recall | - | 2.8% (2.5%-3.0%) | 1.9% (1.8%-2.2%) | 1.4% (1.3%-1.5%) | 1.6% (1.6%-2.4%) |
| | 6 | specificity | - | - | 99.94% (99.94%-99.94%) | 81.89% (67.42%-81.94%) | 81.89% (79.61%-81.95%) |
| | 6 | precision | - | - | 0.1% (0.1%-0.1%) | 0.1% (0.1%-0.1%) | 0.2% (0.2%-0.2%) |
| | 6 | recall | - | - | 0.2% (0.2%-0.3%) | 26.1% (25.8%-45.7%) | 26.4% (25.9%-29.5%) |

## Table S3E Performance summary US-VA, independent training (AUROC)

| Model | Prediction Interval (months) → Exclusion Interval (months) | 0 - 3 | 0 - 6 | 0 - 12 | 0 - 36 | 0 - 60 |
|---|---|---|---|---|---|---|
| Transformer | 0 | 0.899 (0.895-0.903) | 0.857 (0.854-0.862) | 0.825 (0.822-0.828) | 0.775 (0.772-0.778) | 0.762 (0.760-0.765) |
| | 3 | - | 0.783 (0.779-0.786) | 0.761 (0.758-0.764) | 0.758 (0.756-0.761) | 0.745 (0.741-0.747) |
| | 6 | - | - | 0.627 (0.622-0.630) | 0.678 (0.676-0.680) | 0.678 (0.676-0.680) |

## Table S3F Performance summary US-VA, independent training (Specificity/Precision/Recall)

| Model | Exclusion Interval (months) | Prediction Interval (months) → Metric | 0 - 3 | 0 - 6 | 0 - 12 | 0 - 36 | 0 - 60 |
|---|---|---|---|---|---|---|---|
| Transformer | 0 | specificity | 99.96% (99.96%-99.97%) | 99.96% (99.95%-99.96%) | 99.95% (99.94%-99.95%) | 99.91% (99.88%-99.95%) | 99.89% (99.88%-99.91%) |
| | 0 | precision | 11.5% (10.5%-13.2%) | 11.1% (10.4%-12.1%) | 12.0% (10.9%-12.7%) | 8.9% (7.8%-12.2%) | 8.0% (7.6%-9.1%) |
| | 0 | recall | 15.4% (13.4%-16.8%) | 13.0% (11.6%-13.7%) | 10.6% (10.0%-11.3%) | 7.9% (6.6%-9.3%) | 7.3% (6.7%-7.5%) |
| | 3 | specificity | - | 99.95% (99.93%-99.96%) | 99.94% (99.93%-99.97%) | 99.90% (99.89%-99.90%) | 99.90% (99.77%-99.90%) |
| | 3 | precision | - | 2.9% (2.4%-3.7%) | 2.8% (2.4%-4.2%) | 2.9% (2.7%-3.2%) | 2.9% (2.0%-3.2%) |
| | 3 | recall | - | 3.6% (2.8%-4.2%) | 2.8% (2.1%-3.4%) | 2.9% (2.6%-3.1%) | 2.4% (2.2%-3.5%) |
| | 6 | specificity | - | - | 97.18% (96.95%-99.60%) | 96.59% (96.52%-96.99%) | 96.60% (96.52%-97.07%) |
| | 6 | precision | - | - | 0.2% (0.2%-0.2%) | 0.3% (0.3%-0.3%) | 0.3% (0.3%-0.3%) |
| | 6 | recall | - | - | 7.8% (1.2%-8.6%) | 8.3% (7.4%-8.7%) | 7.8% (6.8%-8.3%) |

## Table S3G Performance summary US-VA, independent training above age 50 (AUROC)

| Model | Prediction Interval (months) → Exclusion Interval (months) | 0 - 3 | 0 - 6 | 0 - 12 | 0 - 36 | 0 - 60 |
|---|---|---|---|---|---|---|
| Transformer | 0 | 0.900 (0.895-0.904) | 0.857 (0.853-0.862) | 0.825 (0.821-0.829) | 0.775 (0.772-0.778) | 0.762 (0.760-0.765) |
| | 3 | - | 0.782 (0.778-0.787) | 0.760 (0.758-0.764) | 0.756 (0.754-0.759) | 0.744 (0.741-0.746) |

## Table S3H Performance summary US-VA, independent training above age 50 (Specificity/Precision/Recall)

| Model | Exclusion Interval (months) | Prediction Interval (months) → Metric | 0 - 3 | 0 - 6 | 0 - 12 | 0 - 36 | 0 - 60 |
|---|---|---|---|---|---|---|---|
| Transformer | 0 | specificity | 99.96% (99.96%-99.97%) | 99.95% (99.95%-99.97%) | 99.95% (99.94%-99.95%) | 99.89% (99.88%-99.95%) | 99.89% (99.88%-99.90%) |
| | 0 | precision | 11.8% (11.0%-12.5%) | 11.2% (10.4%-12.7%) | 12.0% (11.2%-12.6%) | 8.2% (7.8%-12.5%) | 8.2% (7.7%-8.6%) |
| | 0 | recall | 15.7% (14.7%-16.9%) | 13.1% (11.5%-13.9%) | 10.8% (10.1%-11.6%) | 8.9% (6.8%-9.4%) | 7.5% (6.9%-7.7%) |
| | 3 | specificity | - | 99.96% (99.95%-99.96%) | 99.96% (99.93%-99.97%) | 99.90% (99.85%-99.90%) | 99.82% (99.81%-99.90%) |
| | 3 | precision | - | 3.6% (2.8%-4.1%) | 3.7% (2.7%-4.6%) | 2.9% (2.5%-3.1%) | 2.3% (2.1%-3.1%) |
| | 3 | recall | - | 3.4% (3.1%-4.0%) | 2.5% (2.2%-3.2%) | 2.9% (2.7%-3.4%) | 2.9% (2.3%-3.2%) |

## Table S3I Performance summary DNPR above age 50 (AUROC)

| Model | Prediction Interval (months): -> | 0-3 | 0-6 | 0-12 | 0-36 | 0-60 |
|---|---|---|---|---|---|---|
| | Exclusion Interval (months) | | | | | |
| Bag-of-words | 0 | 0.600 (0.594-0.605) | 0.611 (0.606-0.615) | 0.622 (0.618-0.627) | 0.621 (0.618-0.624) | 0.605 (0.603-0.608) |
| | 3 | - | 0.646 (0.636-0.656) | 0.653 (0.646-0.660) | 0.633 (0.629-0.637) | 0.609 (0.606-0.612) |
| | 6 | - | - | 0.655 (0.648-0.662) | 0.631 (0.626-0.635) | 0.605 (0.601-0.609) |
| MLP | 0 | 0.758 (0.752-0.763) | 0.747 (0.742-0.751) | 0.727 (0.723-0.731) | 0.690 (0.687-0.694) | 0.665 (0.662-0.668) |
| | 3 | - | 0.686 (0.676-0.696) | 0.672 (0.666-0.680) | 0.662 (0.659-0.666) | 0.644 (0.641-0.648) |
| | 6 | - | - | 0.654 (0.645-0.662) | 0.642 (0.639-0.646) | 0.631 (0.628-0.635) |
| GRU | 0 | 0.854 (0.850-0.858) | 0.827 (0.823-0.831) | 0.777 (0.774-0.781) | 0.717 (0.714-0.720) | 0.686 (0.683-0.690) |
| | 3 | - | 0.733 (0.725-0.744) | 0.710 (0.703-0.715) | 0.667 (0.663-0.671) | 0.645 (0.641-0.648) |
| | 6 | - | - | 0.694 (0.686-0.703) | 0.652 (0.648-0.656) | 0.629 (0.625-0.633) |
| | 12 | - | - | - | 0.629 (0.622-0.634) | 0.610 (0.606-0.614) |
| Transformer | 0 | 0.876 (0.872-0.880) | 0.853 (0.850-0.857) | 0.821 (0.819-0.825) | 0.753 (0.750-0.756) | 0.717 (0.714-0.720) |
| | 3 | - | 0.739 (0.728-0.746) | 0.722 (0.715-0.727) | 0.674 (0.671-0.678) | 0.651 (0.648-0.654) |
| | 6 | - | - | 0.661 (0.655-0.668) | 0.646 (0.642-0.650) | 0.623 (0.620-0.627) |
| | 12 | - | - | - | 0.638 (0.632-0.643) | 0.615 (0.611-0.620) |
| Transformer - Prior knowledge disease | 0 | 0.664 (0.659-0.671) | 0.669 (0.665-0.675) | 0.669 (0.664-0.673) | 0.638 (0.635-0.641) | 0.626 (0.623-0.628) |

## Table S3J Performance summary DNPR above age 50 (Specificity/Precision/Recall)

| Model | | Prediction Interval (months): -> | 0-3 | 0-6 | 0-12 | 0-36 | 0-60 |
|---|---|---|---|---|---|---|---|
| | Exclusion Interval (months) | Metric | | | | | |
| Bag-of-words | 0 | specificity | 96.05% (90.42%-97.10%) | 95.10% (92.06%-96.09%) | 95.82% (92.13%-96.33%) | 90.42% (88.67%-94.49%) | 89.09% (87.19%-90.67%) |
| | 0 | precision | 0.4% (0.3%-0.4%) | 0.5% (0.4%-0.5%) | 0.7% (0.6%-0.7%) | 0.9% (0.9%-1.0%) | 1.0% (1.0%-1.1%) |
| | 0 | recall | 7.3% (5.5%-16.3%) | 9.6% (7.6%-15.1%) | 8.5% (7.4%-14.9%) | 17.1% (10.2%-19.7%) | 17.4% (15.0%-20.2%) |
| | 3 | specificity | - | 99.79% (99.54%-99.80%) | 99.32% (95.53%-99.52%) | 94.59% (88.20%-95.38%) | 87.88% (85.36%-93.80%) |
| | 3 | precision | - | 0.2% (0.2%-0.3%) | 0.4% (0.3%-0.5%) | 0.6% (0.6%-0.7%) | 0.8% (0.7%-0.8%) |
| | 3 | recall | - | 1.0% (0.7%-1.8%) | 2.2% (1.5%-10.5%) | 10.5% (9.0%-21.7%) | 19.6% (10.6%-23.5%) |
| | 6 | specificity | - | - | 99.36% (96.63%-99.39%) | 94.91% (89.44%-99.33%) | 89.80% (85.63%-94.72%) |
| | 6 | precision | - | - | 0.3% (0.2%-0.3%) | 0.5% (0.5%-0.8%) | 0.7% (0.6%-0.7%) |
| | 6 | recall | - | - | 2.2% (1.7%-8.9%) | 9.7% (1.7%-19.5%) | 16.1% (8.8%-22.2%) |
| MLP | 0 | specificity | 99.65% (99.42%-99.68%) | 99.65% (99.42%-99.68%) | 99.64% (99.43%-99.67%) | 99.43% (99.08%-99.52%) | 99.10% (98.88%-99.26%) |
| | 0 | precision | 3.6% (3.1%-4.0%) | 4.6% (3.9%-4.9%) | 5.5% (4.6%-5.9%) | 5.6% (4.6%-6.3%) | 5.0% (4.6%-5.5%) |
| | 0 | recall | 7.9% (7.1%-10.9%) | 7.9% (7.1%-10.1%) | 7.3% (6.8%-8.9%) | 7.2% (6.4%-9.5%) | 7.8% (6.8%-8.8%) |
| | 3 | specificity | - | 99.76% (99.74%-99.80%) | 99.72% (99.47%-99.73%) | 99.08% (98.81%-99.53%) | 98.81% (98.43%-99.01%) |
| | 3 | precision | - | 0.7% (0.5%-0.8%) | 1.3% (1.0%-1.5%) | 1.7% (1.5%-2.2%) | 1.8% (1.6%-1.9%) |
| | 3 | recall | - | 3.4% (2.7%-4.6%) | 3.2% (2.8%-4.9%) | 5.2% (3.2%-6.2%) | 5.0% (4.4%-6.1%) |
| | 6 | specificity | - | - | 99.63% (99.62%-99.66%) | 99.62% (99.25%-99.86%) | 99.55% (98.89%-99.63%) |
| | 6 | precision | - | - | 0.4% (0.3%-0.5%) | 1.3% (1.0%-2.5%) | 1.5% (1.1%-1.9%) |
| | 6 | recall | - | - | 2.0% (1.6%-2.5%) | 2.0% (1.2%-3.2%) | 1.9% (1.5%-3.1%) |
| GRU | 0 | specificity | 99.90% (99.83%-99.91%) | 99.82% (99.78%-99.87%) | 99.77% (99.73%-99.81%) | 99.58% (99.55%-99.68%) | 99.55% (99.53%-99.58%) |
| | 0 | precision | 17.9% (14.0%-19.2%) | 14.8% (13.4%-17.8%) | 14.7% (13.2%-16.5%) | 12.2% (11.6%-13.6%) | 12.1% (11.6%-12.9%) |
| | 0 | recall | 13.0% (12.3%-15.9%) | 14.2% (12.2%-15.9%) | 13.4% (12.4%-14.5%) | 12.1% (11.0%-12.8%) | 10.2% (9.7%-10.6%) |
| | 3 | specificity | - | 99.94% (99.87%-99.95%) | 99.88% (99.84%-99.91%) | 99.71% (99.70%-99.76%) | 99.70% (99.56%-99.71%) |
| | 3 | precision | - | 3.9% (2.7%-4.6%) | 6.4% (5.4%-7.5%) | 6.4% (6.0%-7.1%) | 6.9% (5.6%-7.4%) |
| | 3 | recall | - | 5.4% (4.4%-8.5%) | 6.9% (5.9%-8.0%) | 6.4% (5.8%-6.9%) | 5.1% (4.8%-5.9%) |
| | 6 | specificity | - | - | 99.88% (99.76%-99.91%) | 99.75% (99.69%-99.87%) | 99.55% (99.39%-99.72%) |
| | 6 | precision | - | - | 2.3% (1.8%-2.9%) | 4.3% (3.8%-6.3%) | 3.9% (3.3%-5.1%) |
| | 6 | recall | - | - | 4.4% (3.4%-6.4%) | 4.6% (3.5%-5.1%) | 4.5% (3.6%-5.3%) |
| | 12 | specificity | - | - | - | 99.29% (98.96%-99.47%) | 99.23% (98.60%-99.63%) |
| | 12 | precision | - | - | - | 1.0% (0.9%-1.2%) | 1.3% (1.1%-1.7%) |
| | 12 | recall | - | - | - | 4.0% (3.2%-5.3%) | 3.2% (2.0%-4.8%) |
| Transformer | 0 | specificity | 99.85% (99.83%-99.91%) | 99.85% (99.81%-99.86%) | 99.82% (99.80%-99.86%) | 99.75% (99.72%-99.78%) | 99.72% (99.62%-99.75%) |
| | 0 | precision | 18.1% (16.5%-23.4%) | 20.3% (18.1%-21.5%) | 20.9% (19.7%-23.6%) | 19.1% (17.9%-20.9%) | 18.7% (15.9%-20.0%) |
| | 0 | recall | 18.6% (15.6%-20.0%) | 17.5% (16.4%-19.1%) | 15.9% (14.8%-16.6%) | 12.5% (11.7%-13.1%) | 10.5% (10.0%-11.7%) |
| | 3 | specificity | - | 99.82% (99.79%-99.96%) | 99.82% (99.81%-99.84%) | 99.72% (99.69%-99.80%) | 99.13% (98.98%-99.22%) |
| | 3 | precision | - | 1.8% (1.4%-3.1%) | 4.5% (4.0%-4.9%) | 5.6% (5.2%-6.7%) | 2.7% (2.5%-2.9%) |
| | 3 | recall | - | 6.2% (2.5%-7.7%) | 6.9% (6.2%-7.5%) | 5.5% (4.8%-5.8%) | 5.6% (5.3%-6.2%) |
| | 6 | specificity | - | - | 98.98% (98.96%-99.03%) | 98.83% (98.74%-98.86%) | 98.46% (90.54%-98.63%) |
| | 6 | precision | - | - | 0.2% (0.2%-0.3%) | 0.7% (0.7%-0.8%) | 0.8% (0.7%-0.9%) |
| | 6 | recall | - | - | 3.1% (2.4%-3.7%) | 3.3% (3.0%-3.6%) | 3.4% (3.0%-16.5%) |
| | 12 | specificity | - | - | - | 98.83% (98.82%-98.84%) | 98.74% (98.73%-98.80%) |
| | 12 | precision | - | - | - | 0.5% (0.5%-0.6%) | 0.7% (0.7%-0.8%) |
| | 12 | recall | - | - | - | 3.3% (3.0%-3.6%) | 2.9% (2.6%-3.2%) |
| Transformer - Prior knowledge disease | 0 | specificity | 99.91% (99.79%-99.92%) | 99.80% (99.78%-99.82%) | 99.79% (99.78%-99.80%) | 99.68% (99.65%-99.70%) | 99.67% (99.33%-99.69%) |
| | 0 | precision | 12.0% (7.5%-13.0%) | 9.5% (8.9%-10.4%) | 10.6% (10.1%-11.1%) | 3.6% (3.3%-4.0%) | 3.6% (2.4%-4.0%) |
| | 0 | recall | 7.3% (6.7%-10.5%) | 9.8% (8.9%-10.3%) | 8.8% (8.4%-9.3%) | 2.6% (2.4%-2.8%) | 2.2% (2.0%-2.9%) |

**Supplementary table 4. Known risk factor disease codes.**

A subset of 23 diseases (subset of the 2000 level 3 ICD codes) that have been considered as risk factors for pancreatic cancer [2,3] were chosen for the "known risk factor" analysis. Indeed, most of these are flagged by the IG feature extraction method to make a significant contribution to the machine learning prediction of cancer occurrence (Figure 4). These risk factors were used to train a separate time-series model 'Transformer - known risk factors' for comparison to the model trained on all ICD codes (Figure 3).

| ICD codes | Diseases |
|---|---|
| C18 | Malignant neoplasm of colon |
| C34 | Malignant neoplasm of bronchus and lung |
| C50 | Malignant neoplasm of breast. |
| C61 | Malignant neoplasm of prostate |
| E10, E11 | Type I/II diabetes mellitus |
| E66 | Obesity |
| E78 | High Cholestrol |
| E84 | Cystic fibrosis |
| F32 | Depression |
| I10 | Hypertension |
| I82 | Venous embolism and thrombosis |
| K05 | Periodontal disease |
| K21 | GERD |
| K27 | Peptic Ulcer Disease |
| K50, K51, K52 | Inflammatory bowel disease |
| K85 | Acute Pancreatitis |
| K86 | Chronic Pancreatitis |
| R17 | Jaundice |
| R63 | Weight loss |
| Z92 | Personal history of medical treatment |

**Supplementary table 5. Disease attribution after training with 3 months data exclusion - Denmark DNPR & US-VA**

In order to discover the top diseases that contribute to our model's risk prediction, we calculated the contribution score for all input features using integrated gradients (IG), an attribution method for neural networks. The IG contribution score (arbitrary units) was calculated for trajectories with cancer occurrence in the time windows 0-6 months, 6-12 months, 12-24 months and 24-36 months for DNPR and US-VA data, both with 3 months data exclusion. - A list with features without data exclusion is in Figure 5. - We mapped ICD9 diagnosis codes to ICD10 for the US-VA dataset to keep the description comparable, and therefore multiple code descriptions might be listed for a given ICD9 code.

**DNPR - Denmark Healthcare system**

Disease contribution to the risk assessment of cancer occurrence at different times between assessment and actual occurrence of cancer (DK-DNPR)

Diseases contribution at different time to cancer (DNPR)

| Cancer in 0-6 months | Cancer in 6-12 months | Cancer in 12-24 months | Cancer in 24-36 months |
|---|---|---|---|
| Other diseases of biliary tract (32.3335) | Other diseases of biliary tract (25.4905) | Other diseases of biliary tract (26.2387) | Non-insulin-dependent diabetes mell itus (11.9299) |
| Unspecified jaundice (14.3137) | Other diseases of pancreas (11.5739) | Non-insulin-dependent diabetes mell itus (17.4123) | Other diseases of biliary tract (11.2389) |
| Other diseases of pancreas (13.5165) | Unspecified jaundice (10.1354) | Medical observation and evaluation for suspected diseases and conditions (13.7912) | Other diseases of pancreas (8.8495) |
| Non-insulin-dependent diabetes mell itus (9.1564) | Non-insulin-dependent diabetes mell itus (8.7353) | Other diseases of pancreas (11.5773) | Medical observation and evaluation for suspected diseases and conditions (8.5102) |
| Diseases of pancreas (8.8114) | Medical observation and evaluation for suspected diseases and conditions (7.5375) | Abdominal and pelvic pain (4.8105) | Unspecified jaundice (4.2823) |
| Abdominal and pelvic pain (8.1039) | Diseases of pancreas (5.4421) | Diseases of pancreas (4.2698) | Benign neoplasm of colon, rectum, a nus and anal canal (3.2988) |
| Acute pancreatitis (5.7806) | Abdominal and pelvic pain (3.3334) | Acute pancreatitis (3.2563) | Abdominal and pelvic pain (3.1899) |
| Malignant neoplasm of stomach (4.6699) | Malignant neoplasm of bronchus and lung (2.2486) | Unspecified jaundice (2.9892) | Gastritis and duodenitis (2.8434) |
| Medical observation and evaluation for suspected diseases and conditions (3.6176) | Benign neoplasm of colon, rectum, a nus and anal canal (2.1298) | Benign neoplasm of colon, rectum, a nus and anal canal (2.7481) | Gingivitis and periodontal diseases (2.7876) |
| Other anaemias (3.1611) | Diabetes mellitus (1.9986) | Other anaemias (2.5468) | Malignant neoplasm of bronchus and lung (2.4107) |
| Diabetes mellitus (2.5442) | Abnormal involuntary movements (1.6557) | Gastro-oesophageal reflux disease (2.2908) | Gastro-oesophageal reflux disease (1.9136) |
| Gastro-oesophageal reflux disease (2.4501) | Other anaemias (1.6202) | Disorders of sphingolipid metabolis m and other lipid storage disorders (2.0459) | Acute pancreatitis (1.7894) |
| Dyspepsia (2.1679) | Other symptoms and signs involving the digestive system and abdomen (1.5917) | Malignant neoplasm of bronchus and lung (1.9628) | Malignant neoplasm of other and uns pecified parts of biliary tract (1.6697) |
| Bacterial pneumonia, not elsewhere classified (2.0704) | Gastritis and duodenitis (1.5842) | Diabetes mellitus (1.8423) | Other anaemias (1.5393) |
| Malignant neoplasm of bronchus and lung (1.6351) | Cholelithiasis (1.4921) | Enlarged lymph nodes (1.7293) | Diabetes mellitus (1.2959) |
| Cholelithiasis (1.5319) | Gastro-oesophageal reflux disease (1.4884) | Other intervertebral disc disorders (1.6947) | Angina pectoris (1.2408) |
| Benign neoplasm of colon, rectum, a nus and anal canal (1.3892) | Secondary malignant neoplasm of res piratory and digestive organs (1.4277) | Bacterial pneumonia, not elsewhere classified (1.5436) | Dyspepsia (1.0569) |
| Dislocation, sprain and strain of j oints and ligaments of head (1.3044) | Mental and behavioural disorders du e to use of tobacco (1.416) | Gastritis and duodenitis (1.4928) | Malignant neoplasm of stomach (1.0218) |
| Malignant neoplasm of small intesti ne (1.2895) | Malignant neoplasm of stomach (1.4045) | Other functional intestinal disorde rs (1.4278) | Diseases of pancreas (1.0155) |
| Pneumonia, organism unspecified (1.1685) | Osteoporosis without pathological f racture (1.3343) | Dyspepsia (1.4028) | Mental and behavioural disorders du e to use of tobacco (0.9639) |
| Osteoporosis without pathological f racture (1.1565) | Other diseases of gallbladder and b iliary (1.2574) | Delirium, not induced by alcohol an d other psychoactive substances (1.1866) | Delirium, not induced by alcohol an d other psychoactive substances (0.9083) |
| Other symptoms and signs involving the digestive system and abdomen (1.1477) | Acute pancreatitis (1.1292) | Hyperparathyroidism and other disor ders of parathyroid gland (1.164) | Other intervertebral disc disorders (0.8991) |
| Malignant neoplasm of other and uns pecified parts of biliary tract (1.1396) | Dyspepsia (1.1197) | Insulin-dependent diabetes mellitus (1.1136) | Disorders of pancreatic internal se cretion other than diabetes mellitus (0.895) |
| Malignant neoplasm without specific ation of site (1.133) | Bacterial pneumonia, not elsewhere classified (1.0645) | Chronic ulcer of skin (1.087) | Dislocation, sprain and strain of j oints and ligaments of shoulder girdle (0.8586) |

| | | | |
|---|---|---|---|
| Sequelae of poisoning by drugs, med icaments and biological substances (1.087) | Aortic aneurysm and dissection (1.0609) | Malignant neoplasm of stomach (1.0713) | Bacterial pneumonia, not elsewhere classified (0.8422) |
| Gastritis and duodenitis (1.0649) | Dislocation, sprain and strain of j oints and ligaments of shoulder girdle (0.8825) | Postprocedural respiratory disorder s, not elsewhere classified (1.0706) | Open wound of wrist and hand (0.8262) |
| Umbilical hernia (1.049) | Enlarged lymph nodes (0.7821) | Cholelithiasis (1.0693) | Special screening examination for n eoplasms (0.8235) |
| Malignant neoplasm of cervix uteri (0.9971) | Postprocedural respiratory disorder s, not elsewhere classified (0.7585) | Secondary malignant neoplasm of res piratory and digestive organs (0.9917) | Insulin-dependent diabetes mellitus (0.8152) |
| Noninflammatory disorders of ovary, fallopian tube and broad ligament (0.974) | 850 (0.6887) | Benign mammary dysplasia (0.9914) | Paralytic ileus and intestinal obst ruction without hernia (0.8109) |

| | | | |
|---|---|---|---|
| Insulin-dependent diabetes mellitus (0.9269) | Other noninflammatory disorders of vulva and perineum (0.6836) | Gingivitis and periodontal diseases (0.9797) | Observation, without need for furth er medical care (0.7409) |
| Secondary malignant neoplasm of res piratory and digestive organs (0.9135) | Chronic ulcer of skin (0.6428) | Other chronic obstructive pulmonary disease (0.9633) | Acute myocardial infarction (0.7025) |
| Other noninflammatory disorders of vulva and perineum (0.865) | Dislocation, sprain and strain of j oint and ligaments of hip (0.6387) | Aortic aneurysm and dissection (0.9152) | Obesity (0.6952) |
| Mental and behavioural disorders du e to use of tobacco (0.8545) | Dislocation, sprain and strain of j oints and ligaments of head (0.6196) | Paralytic ileus and intestinal obst ruction without hernia (0.8735) | Personal history of malignant neopl asm (0.6901) |
| 850 (0.8509) | Other cerebrovascular diseases (0.6025) | Osteoporosis without pathological f racture (0.8014) | Other diseases of oesophagus (0.6649) |
| Delirium, not induced by alcohol an d other psychoactive substances (0.7508) | Malignant neoplasm without specific ation of site (0.5877) | Malignant neoplasm of other and uns pecified parts of biliary tract (0.8013) | Dislocation, sprain and strain of j oints and ligaments at ankle and foot level (0.6579) |
| Malignant neoplasm of gallbladder a nd bile ducts (0.7488) | Chronic renal failure (0.5764) | Disorders of globe (0.7984) | Benign neoplasm of urinary organs (0.6276) |
| Mental and behavioural disorders du e to use of alcohol (0.7157) | Malignant neoplasm of other and uns pecified parts of biliary tract (0.5745) | 850 (0.794) | Dislocation, sprain and strain of j oints and ligaments at wrist and hand level (0.6254) |
| Complications and misadventures in operative therapeutic procedures (0.685) | Acute myocardial infarction (0.5735) | Open wound of wrist and hand (0.7659) | Hypotension (0.6154) |
| Enlarged lymph nodes (0.6249) | Malignant neoplasm of gallbladder a nd bile ducts (0.5688) | Neoplasm of unspecified nature of d igestive organs (0.7392) | Cerebral infarction (0.6125) |
| Other diseases of gallbladder and b iliary (0.6058) | Gastric ulcer (0.5565) | Other septicaemia (0.717) | Disorders of sphingolipid metabolis m and other lipid storage disorders (0.5905) |
| Phlebitis and thrombophlebitis (0.5884) | Other chronic obstructive pulmonary disease (0.5372) | Symptomatic heart disease (0.7164) | Cutaneous abscess, furuncle and car buncle (0.5888) |
| Benign neoplasm of other and ill-de fined parts of digestive system (0.5648) | Synovitis and tenosynovitis (0.5352) | Mental and behavioural disorders du e to use of tobacco (0.6664) | Transient cerebral ischaemic attack s and related syndromes (0.5777) |
| Other venous embolism and thrombosi s (0.5452) | Convulsions, not elsewhere classifi ed (0.519) | Abnormal involuntary movements (0.6605) | Cholelithiasis (0.5719) |
| Acute myocardial infarction (0.5372) | Other diseases of oesophagus (0.5127) | Diseases of vocal cords and larynx, not elsewhere classified (0.6412) | Aortic aneurysm and dissection (0.5665) |
| Other surgical follow-up care (0.5349) | Other coagulation defects (0.512) | Other symptoms and signs involving the digestive system and abdomen (0.6085) | Other disorders of bone density and structure (0.5537) |
| Other noninfective gastroenteritis and colitis (0.5322) | Obesity (0.5105) | Dislocation, sprain and strain of j oints and ligaments of head (0.6056) | Unspecified diabetes mellitus (0.5417) |
| Unspecified acute lower respiratory infection (0.5144) | Disorders of sphingolipid metabolis m and other lipid storage disorders (0.4908) | Hypotension (0.5991) | Phlebitis and thrombophlebitis (0.5181) |
| Other diseases of oesophagus (0.5117) | Heart failure (0.4866) | 825 (0.5963) | Synovitis and tenosynovitis (0.502) |
| Gastro-enteritis and colitis, excep t ulcerative, of non-infectious origin (0.4643) | Alcoholic liver disease (0.4693) | Atrial fibrillation and flutter (0.5832) | Other diseases of intestine (0.4841) |
| Malignant neoplasm of connective an d other soft tissue (0.4607) | None (0.4646) | Chronic diseases of tonsils and ade noids (0.5745) | Umbilical hernia (0.4795) |

## US-VA  Healthcare system

Disease contribution to the risk assessment of cancer occurrence at different times between assessment and actual occurrence of cancer (US-VA)

| Cancer in 0-6 months | Cancer in 6-12 months | Cancer in 12-24 months | Cancer in 24-36 months |
|---|---|---|---|
| Acute pancreatitis<br>Other diseases of pancreas | Non-insulin-dependent diabetes mellitus<br>Insulin-dependent diabetes mellitus<br>Other specified diabetes mellitus | Non-insulin-dependent diabetes mellitus<br>Insulin-dependent diabetes mellitus<br>Other specified diabetes mellitus | Mental and behavioral disorders due to psychoactive substances use |
| Non-insulin-dependent diabetes mellitus<br>Insulin-dependent diabetes mellitus<br>Other specified diabetes mellitus | Mental and behavioral disorders due to psychoactive substances use | Mental and behavioral disorders due to psychoactive substances use | Non-insulin-dependent diabetes mellitus<br>Insulin-dependent diabetes mellitus<br>Other specified diabetes mellitus |
| Other symptoms and signs involving the digestive system and abdomen | Persons encountering health services in other circumstances | Persons encountering health services in other circumstances | Persons encountering health services in other circumstances |
| Persons encountering health services in other circumstances | Other symptoms and signs involving the digestive system and abdomen | Other arthritis | Benign neoplasm of digestive system |
| Mental and behavioral disorders due to psychoactive substances use | Other arthritis | Other symptoms and signs involving the digestive system and abdomen | Symptoms and signs involving the digestive system and abdomen |
| Other diseases of biliary tract | Mental and behavioral disorders due to psychoactive substances use | Symptoms and signs involving the digestive system and abdomen | Malignant neoplasm of prostate |
| Symptoms and signs involving the digestive system and abdomen | Benign neoplasm of digestive system | Benign neoplasm of digestive system | Symptoms and signs involving the circulatory and respiratory systems |
| Benign neoplasm of digestive system | Other diseases of biliary tract | Malignant neoplasm of prostate | Other arthritis |
| Other arthritis | Malignant neoplasm of prostate | Mental and behavioral disorders due to psychoactive substances use | Other symptoms and signs involving the digestive system and abdomen |
| Symptoms and signs involving the circulatory and respiratory systems | Symptoms and signs involving the circulatory and respiratory systems | Abnormal findings on examination of blood, without diagnosis<br>Other disorders of thyroid | Abnormal findings on examination of blood, without diagnosis<br>Other disorders of thyroid |
| Other diseases of pancreas | Symptoms and signs involving the digestive system and abdomen | Symptoms and signs involving the circulatory and respiratory systems | Age-related cataract and Other cataract |
| Mental and behavioral disorders due to psychoactive substances use | Acute pancreatitis<br>Other diseases of pancreas | Other diseases of biliary tract | Glaucoma |
| Age-related cataract and Other cataract | Viral hepatitis | Acute pancreatitis<br>Other diseases of pancreas | Viral hepatitis |
| Viral hepatitis | Essential (primary) hypertension<br>I169 | Radiation-related disorders of the skin and subcutaneous tissue | Mental and behavioral disorders due to psychoactive substances use |
| Malignant neoplasm of prostate | Abnormal findings on examination of blood, without diagnosis<br>Other disorders of thyroid | Age-related cataract and Other cataract | Radiation-related disorders of the skin and subcutaneous tissue |

| | | | |
|---|---|---|---|
| Long term (current) drug therapy<br>Encounters for other specific health care | Cellulitis | Viral hepatitis | Other disorders of urinary system |
| Special screening examination for other diseases and disorders | papulosquamous disorders | Other disorders of the skin and subcutaneous tissue | Other diseases of biliary tract |
| Symptoms and signs involving the skin and subcutaneous tissue | Non-insulin-dependent diabetes mellitus | Other disorders of urinary system | Cellulitis |
| Abnormal findings on examination of blood, without diagnosis<br>Other disorders of thyroid | Age-related cataract and Other cataract | Follow-up examination after treatment for malignant neoplasms and others | Acute pancreatitis<br>Other diseases of pancreas |
| Cellulitis | Other diseases of pancreas | Symptoms and signs involving the skin and subcutaneous tissue<br>Oedema, not elsewhere classified<br>Unspecified jaundice | Other disorders of the skin and subcutaneous tissue |
| Special screening examination for other diseases and disorders | Radiation-related disorders of the skin and subcutaneous tissue | Glaucoma | Other spondylopathies<br>Dorsalgia<br>Other dorsopathies, not elsewhere classified |
| Essential (primary) hypertension<br>Hypertension Crisis | Symptoms and signs involving the skin and subcutaneous tissue<br>Oedema, not elsewhere classified<br>Unspecified jaundice | Dermatitis and eczema | Dermatitis and eczema |
| Overweight and obesity | Dermatophytosis | Special screening examination for other diseases and disorders | Cervical disc disorders<br>Other intervertebral disc disorders<br>Postprocedural musculoskeletal disorders, not elsewhere classified |
| Symptoms and signs concerning food and fluid intake | Special screening examination for other diseases and disorders | Other spondylopathies<br>Dorsalgia<br>Other dorsopathies, not elsewhere classified | Disorders of skin appendages |
| Other spondylopathies<br>Dorsalgia<br>Other dorsopathies, not elsewhere classified | Other spondylopathies<br>Dorsalgia<br>Other dorsopathies, not elsewhere classified | Heart disease | Symptoms and signs involving the skin and subcutaneous tissue<br>Oedema, not elsewhere classified<br>Unspecified jaundice |
| Paroxysmal tachycardia<br>Atrial fibrillation and flutter<br>Other cardiac arrhythmias<br>Cardiac arrest<br>Abnormalities of heart beat | Follow-up examination after treatment for malignant neoplasms and others | Cellulitis | Special screening examination for other diseases and disorders |
| Presence of other functional implants | Glaucoma | Diverticular disease of intestine | Heart disease |
| Other medical care | Other medical care | Encounters for other specific health care<br>Long term (current) drug therapy | Follow-up examination after treatment for malignant neoplasms and others |
| Glaucoma | Other disorders of urinary system | papulosquamous disorders | Presence of other devices<br>And other functional implants |
| Dermatophytosis | Other disorders of the skin and subcutaneous tissue | Other benign neoplasms of skin | Persons with potential health hazards related to socioeconomic and psychosocial circumstances |

| | | | |
|---|---|---|---|
| Follow-up examination after treatment for malignant neoplasms and others | Dermatitis and eczema | Cervical disc disorders<br>Other intervertebral disc disorders<br>Postprocedural musculoskeletal disorders, not elsewhere classified | Acquired deformities of fingers and toes |
| Other diseases of the urinary system | Cervical disc disorders<br>Other intervertebral disc disorders<br>Postprocedural musculoskeletal disorders, not elsewhere classified | Persons with potential health hazards related to socioeconomic and psychosocial circumstances | Other forms of heart disease |
| Symptoms and signs involving the skin and subcutaneous tissue<br>Edema, not elsewhere classified<br>Unspecified jaundice | Heart disease | Radiation-related disorders of the skin and subcutaneous tissue | Mental and behavioural disorders due to use of alcohol |
| Papulosquamous disorders | Persons with potential health hazards related to socioeconomic and psychosocial circumstances | Other malignant neoplasms of skin | Other malignant neoplasms of skin |
| Other disorders of the skin and subcutaneous tissue | Cholelithiasis | Acquired deformities of fingers and toes | Other benign neoplasms of skin |
| Other forms of heart disease | Symptoms and signs concerning food and fluid intake | Dermatophytosis | Diverticular disease of intestine |
| Diverticular disease of intestine | Other forms of heart disease | Other forms of heart disease | Nail disorders |
| Persons with potential health hazards related to socioeconomic and psychosocial circumstances | Corns and callosities | Pulmonary disease<br>Chronic bronchitis | Varicose veins of lower extremities |
| Dermatitis and eczema | Diverticular disease of intestine | Mental and behavioral disorders due to use of alcohol | Symptoms and signs concerning food and fluid intake |
| Disorders of refraction and accommodation | Presence of other devices<br>And other functional implants | Other diseases of digestive system | Other disorders of male genital organs |
| Cholecystitis<br>Other diseases of gallbladder | Other diseases of digestive system | Varicose veins of lower extremities | Asthma<br>Other chronic obstructive pulmonary disease |
| Other benign neoplasms of skin | Overweight and obesity | Disorders of arteries, arterioles and capillaries in diseases classified elsewhere | Calculus of kidney and ureter |
| Varicose veins of lower extremities | Other disorders of male genital organs | Personal history of other diseases and conditions | Disorders of choroid and retina |
| Malignant neoplasm of trachea<br>Malignant neoplasm of bronchus and lung | Acquired deformities of fingers and toes | Presence of other devices<br>And other functional implants | Herpesviral [herpes simplex] infections |
| Cholelithiasis | Disorders of arteries, arterioles and capillaries in diseases classified elsewhere | Other cerebrovascular diseases | Other venous embolism and thrombosis |
| Disorders of skin appendages | Other benign neoplasms of skin | Nail disorders | Dermatophytosis |

| | | | |
|---|---|---|---|
| Non-insulin-dependent diabetes mellitus | Encounters for other specific health care<br>Long term (current) drug therapy | Other disorders of male genital organs | Angina pectoris |
| Mental and behavioral disorders due to use of alcohol | Nonrheumatic aortic valve disorders | Heart failure | Disorders of arteries, arterioles and capillaries in diseases classified elsewhere |
| Disorders of arteries, arterioles and capillaries in diseases classified elsewhere | Disorders of skin appendages | Non-insulin-dependent diabetes mellitus | Disorders of external ear in diseases classified elsewhere |
| Other disorders of male genital organs | Mononeuropathies of upper limb<br>Other mononeuropathies | Disorders of external ear in diseases classified elsewhere | Inguinal hernia |

## Supplementary table 6: Checklist for supervised clinical ML study

| Study design (Part 1) | Completed: | page number | Notes if not completed |
|---|---|---|---|
| The clinical problem in which the model will be employed is clearly detailed in the paper. | Y | | |
| The research question is clearly stated. | Y | | |
| The characteristics of the cohorts (training and test sets) are detailed in the text. | Y | | |
| The cohorts (training and test sets) are shown to be representative of real-world clinical settings. | Y | | |
| The state-of-the-art solution used as a baseline for comparison has been identified and detailed. | Y | | |

| **Data and optimization (Parts 2, 3)** | **Completed: page number** | | **Notes if not completed** |
|---|---|---|---|
| The origin of the data is described and the original format is detailed in the paper. | Y | | |
| Transformations of the data before it is applied to the proposed model are described. | Y | | |
| The independence between training and test sets has been proven in the paper. | Y | | |
| Details on the models that were evaluated and the code developed to select the best model are provided. | Y | | |
| | | | |
| Is the input data type structured or unstructured? | X Structured | | ☐ Unstructured |
| **Model performance (Part 4)** | **Completed: page number** | | **Notes if not completed** |
| The primary metric selected to evaluate algorithm performance (eg: AUC, F-score, etc) including the justification for selection, has been clearly stated. | Y | | |
| The primary metric selected to evaluate the clinical utility of the model (eg PPV, NNT, etc) including the justification for selection, has been clearly stated. | Y | | |
| The performance comparison between baseline and proposed model is presented with the appropriate statistical significance. | Y | | |

| Model Examination (Parts 5) | Completed: | page number | Notes if not completed |
|---|---|---|---|
| Examination Technique 1[a] | Y | | |
| Examination Technique 2[a] | Y | | |
| A discussion of the relevance of the examination results with respect to model/algorithm performance is presented. | Y | | |
| A discussion of the feasibility and significance of model interpretability at the case level if examination methods are uninterpretable is presented. | Y | | |
| A discussion of the reliability and robustness of the model as the underlying data distribution shifts is included. | Y | | |
| *Common examination approaches based on study type:<br>* For studies involving exclusively structured data coefficients and sensitivity analysis are often appropriate<br>* For studies involving unstructured data in the domains of image analysis or NLP: saliency maps (or equivalents) and sensitivity analysis are often appropriate | | | |
| **Reproducibility (Part 6): choose appropriate tier of transparency** | | | **Notes** |
| Tier 1: complete sharing of the code | | X | |

| | | |
|---|---|---|
| Tier 2: allow a third party to evaluate the code for accuracy/fairness; share the results of this evaluation | ☐ | |
| Tier 3: release of a virtual machine (binary) for running the code on new data without sharing its details | ☐ | |
| Tier 4: no sharing | ☐ | |

PPV: Positive Predictive Value

NNT: Numbers Needed to Treat

[a] Common examination approaches based on study type: for studies involving exclusively structured data, coefficients and sensitivity analysis are often appropriate; for studies involving unstructured data in the domains of image analysis or natural language processing, saliency maps (or equivalents) and sensitivity analyses are often appropriate. Select 2 from this list or choose an appropriate technique, document each technique used on the appropriate line above.

## Supplementary notes

## Draft economic considerations for the design of clinical screening trial

Whether or not cancer is actually detected early in high-risk patients of course depends on the frequency and type of clinical tests performed in a surveillance program. Detailed deliberations with clinicians will be required to design the details of such a program, with considerations for minimizing cost and minimizing potential harm from invasive procedures. For cost considerations alone, we provide a hypothetical tradeoff algorithm (below). A surveillance program will also have to be supplemented with counseling, in analogy to genetic counseling, to provide patients with an informed choice and to minimize potential anxiety. The numerical estimates of performance of the risk assessment tool presented here are simply one of several considerations needed for the design of a surveillance program.

We propose a toy estimate of a practical scenario for a screening trial, taking into account typically available real-world data, the accuracy of prediction on such data, the estimated cost of a screening trial, the cost of clinical screening methods and the overall potential benefit of treatment.

The detailed design of a prediction-surveillance program, to be explored in a clinical setting, depends on the organization of a particular health care system. In a ‘walk in’ scenario, in approximate analogy to colonoscopic screening for colorectal cancer, patients older than, e.g., age 50 would be invited for assessment of their risk by the prediction tool on a yearly basis and, if identified as high-risk, offered a series of follow-up visits with extensive clinical testing. In a ‘national system’ scenario, possible in centralized health systems with location-independent centralized aggregation of electronic health records, risk assessment could be done on an ongoing basis, possibly for each patient whenever a new disease event occurs. If a high-risk prediction is triggered, the responsible physician would receive an alert. With a diversity of scenarios, it is reasonable to propose a clinical prediction-surveillance program tailored to the health system in a particular country.

To illustrate the economic benefits of such a screening and to stimulate discussion regarding the optimal design of a prediction-surveillance program, we have made a first-order-estimate for a clinical screening trial of 10,000 people using the best model (the transformer model). For simplicity, we have made no assumptions regarding age distribution. In the calculation below, the cost of running the software on the entire population has been neglected, as it is marginal compared to the rest of the expenses (the algorithm can easily assess the risk using computers available in the hospitals, e.g., in less than an hour of run time on a single computer). Here is a simple economic model.

Net Benefit = Average benefit for each correctly identified cancer patient * TP

− Monitoring expense for each high-risk patient * P

− Basic cost per enrollee * N

where the screening cohort is N=10,000 and TP is the number of true positives, i.e., the number of correctly identified high-risk patients, and P is the number of actual positive patients, which we estimated using cancer incidence of the DNPR dataset. In our cost-benefit estimate, we arbitrarily set the screening trial cost at $200 per enrollee, the additional monitoring expense for a patient predicted at high risk by screening at $10,000 and the extra cost saved for advanced treatment for each monitored patient at $200,000, averaged over those in which cancer is detected (savings in excess of $200,000) and those in which it is not detected (no savings).

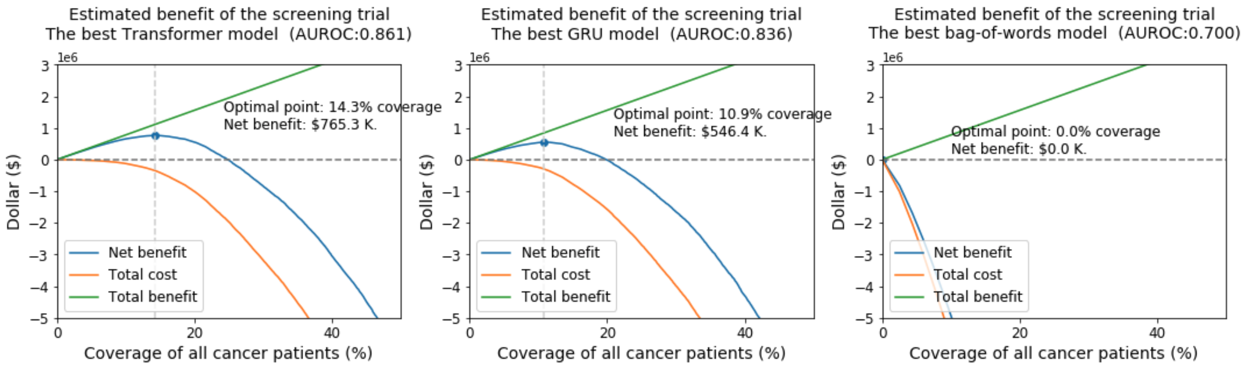


**Estimate of financial benefits for different models.** We analyzed each possible operational point and calculated the corresponding cost and benefit, using ballpark estimates. We plotted the net benefits as a function of coverage of cancer patients, i.e. recall or sensitivity. Covering more cancer patients plausibly leads to a larger total benefit, but the total cost also increases. The optimal point is picked for maximal net benefit.

An optimal decision threshold has to balance the cost of assessment and testing against the potential financial benefit for reducing treatment cost. Using this simplified model, we estimated the net benefits of different models with all possible operational points. Such a screening trial for 10,000 people would have $760,000 net benefit by choosing the balance between true and false positives such that the net benefit is optimal. This corresponds to a precision of 14.0% and a specificity of 99.7%. In contrast, a less good model GRU would have $540K net benefits but a bag-of-words model (baseline) would have no net benefits for any operational point because of the low incidence of pancreatic cancer.

The proposed concrete but hypothetical design of a screening trial is intended to guide the debate and ultimate decisions regarding implementation with clinicians and healthcare professionals. However, this calculation is based on roughly estimated numbers and does not reflect real-world cost analysis. Nor does this economic model

reflect the non-monetary benefits to patients' quality of life, which should be the dominant factor in the design of prediction-surveillance and early intervention programs. In a real-world scenario, clinicians and payers in a particular health system have the opportunity to optimize the design of such trials with realistic cost-benefit parameters, as well as consideration of communication ethics and the non-financial aspects of patient benefit.

A key challenge for future realistic economic estimates is the mapping between ICD (diagnosis) codes to CPT (billing) codes that are used for expense calculations and reimbursements. In addition, in the US, there is substantial geographical variability in reimbursement even for the same CPT/billing codes.

## Comparison with surveillance programs based on family history and germline variants

Interestingly, the relative risk as reported in this study is comparable to that reported for patients with genetic risk factors such as mutations in the BRCA2, ATM, PALB2 and other genes or those with a high value of a polygenic risk score [3,4]; and, those with family history of pancreatic cancer are reported to have a 9x higher risk of pancreatic cancer (relative risk). But these criteria cover only a small fraction of the total population, as genetic or detailed family history data is available only in a relatively small number of patients and heritability is estimated to explain only 4-5% of all pancreatic cancers. In this work, complementary to established surveillance programs for patients with elevated risk based on germline variants or family history, the focus is on the application to the entire real-world patient population. The real-world AI approach casts a much wider net and supports application in practice in that running the prediction program routinely on, say, 1 million patients - is relatively inexpensive (see Result S1). In planning the scope of a realistic surveillance program, in addition to prediction performance, the cost of screening is a key leading factor in determining the fraction of high-risk patients nominated for a screening program in any particular health care system.

## Information contribution as a function of time gap between of assessment and cancer occurrence

The exclusion of trajectories ending very close to pancreatic cancer removes the influence of disease codes that represent late symptoms of pancreatic cancer or are otherwise easily attributable to pancreatic cancer. However, data exclusion of such late events alone does not quantify the influence of longer term risk factors on prediction. We therefore computed the recall rate of prediction as a function of the time-to-cancer, defined as the time between the end of disease trajectory and the occurrence of cancer (Figure 5A, C). As expected, recall levels decrease with longer time-to-cancer, from 8% for cancer occurring about 1 year after assessment to a recall

of 4% for cancer occurring about 3 years after assessment (DNPR, Figure 5A). This suggests that the model not only learns from symptoms very close to pancreatic cancer but also from longer disease history, albeit at lower accuracy.

**Survival curves for DNPR and US-VA datasets.**

For the Danish dataset, five-year survival was 23% for Stage I, 8.3% for Stage II, 2.3% for Stage III, and 0.8 for Stage IV. Median survival was 645 days for Stage I, 483 days for Stage II, 262 days for Stage III, and 81 days for Stage IV.

For the US-VA dataset, five-year survival was 19.4% for Stage I, 8.8% for Stage II, 3.2% for Stage III, and 1.3% for Stage IV. Median survival was 424 days for Stage I, 330 days for Stage II, 243 days for Stage III, and 91 days for Stage IV.

Ordered for comparison:

| *Median survival [days]* | *DNPR* | *US-VA* |
|---|---|---|
| *Stage I* | *645* | *424* |
| *Stage II* | *483* | *330* |
| *Stage III* | *262* | *243* |
| *Stage IV* | *81* | *91* |

The uneven distribution of sex in the VA database may contribute to the differences in survival. The overall similarity of the survival curves in the two datasets adds some confidence to the quality of the data, as selected for the study.

REFERENCES


1. Sasaki, Y. The truth oh the f--measure. *Manchester: School of Computer Science, University of Manchester* (2007).
2. Yuan, C. *et al.* Diabetes, Weight Change, and Pancreatic Cancer Risk. *JAMA Oncol* **6**, e202948 (2020).
3. Klein, A. P. Pancreatic cancer epidemiology: understanding the role of lifestyle and inherited risk factors. *Nat. Rev. Gastroenterol. Hepatol.* (2021) doi:10.1038/s41575-021-00457-x.
4. Galeotti, A. A. *et al.* Polygenic and multifactorial scores for pancreatic ductal adenocarcinoma risk prediction. *J. Med. Genet.* **58**, 369–377 (2021).
