# Supplementary material for: A deep learning algorithm to predict risk of pancreatic cancer from disease trajectories
Source: Nat Med. 2023 May 8;29(5):1113–22. doi: 10.1038/s41591-023-02332-5 (PMC10202814; doi:10.1038/s41591-023-02332-5)
Supplement: Supplementary file 2 — Reporting Summary [file 41591_2023_2332_MOESM2_ESM.pdf]

nature portfolio

Corresponding author(s): Søren Brunak ,Chris Sander

Last updated by author(s): Mar 29, 2023

Reporting Summary

Nature Portfolio wishes to improve the reproducibility of the work that we publish. This form provides structure for consistency and transparency in reporting. For further information on Nature Portfolio policies, see our Editorial Policies and the Editorial Policy Checklist.

Statistics

For all statistical analyses, confirm that the following items are present in the figure legend, table legend, main text, or Methods section.

n/a	Confirmed	
☐	☒	The exact sample size (*n*) for each experimental group/condition, given as a discrete number and unit of measurement
☐	☒	A statement on whether measurements were taken from distinct samples or whether the same sample was measured repeatedly
☒	☐	The statistical test(s) used AND whether they are one- or two-sided<br>*Only common tests should be described solely by name; describe more complex techniques in the Methods section.*
☐	☒	A description of all covariates tested
☒	☐	A description of any assumptions or corrections, such as tests of normality and adjustment for multiple comparisons
☐	☒	A full description of the statistical parameters including central tendency (e.g. means) or other basic estimates (e.g. regression coefficient) AND variation (e.g. standard deviation) or associated estimates of uncertainty (e.g. confidence intervals)
☒	☐	For null hypothesis testing, the test statistic (e.g. *F*, *t*, *r*) with confidence intervals, effect sizes, degrees of freedom and *P* value noted<br>*Give P values as exact values whenever suitable.*
☒	☐	For Bayesian analysis, information on the choice of priors and Markov chain Monte Carlo settings
☐	☒	For hierarchical and complex designs, identification of the appropriate level for tests and full reporting of outcomes
☒	☐	Estimates of effect sizes (e.g. Cohen's *d*, Pearson's *r*), indicating how they were calculated

Our web collection on statistics for biologists contains articles on many of the points above.

Software and code

Policy information about availability of computer code

Data collection	No software was used for data collection.
Data analysis	The software, under the name CancerRiskNet (initial commit Feb 22, 2023), is freely available at https://github.com/BrunakSanderLabs/CancerRiskNet github repository. All the analysis carried out in the study were performed using python v3.8.10. The python package torch vl.9.0 was used for developing the machine learning models; Integrated gradient was computed using the python package Captum v0.3.1. Visualization was obtained using python packages matplotlib v3.3.2 and seaborn v0.11.0.

For manuscripts utilizing custom algorithms or software that are central to the research but not yet described in published literature, software must be made available to editors and reviewers. We strongly encourage code deposition in a community repository (e.g. GitHub). See the Nature Portfolio guidelines for submitting code & software for further information.

Data

Policy information about availability of data

All manuscripts must include a data availability statement. This statement should provide the following information, where applicable:

- Accession codes, unique identifiers, or web links for publicly available datasets
- A description of any restrictions on data availability
- For clinical datasets or third party data, please ensure that the statement adheres to our policy

Danish registry-based studies do not require ethical approvals, and informed consent is not required. The study has been approved by the Danish Health Data

Authority (FSEID-00003092 and FSEID-00004491), the Danish Data Protection Agency (ref: SUND-2017-57) and GDPR record of processing activity (ref: 514-0255/18-3000). Application for data access can be made to the Danish Health Data Authority (contact: servicedesk@sundhedsdata.dk). Anyone wishing access to the data and use them for research will be required to meet research credentialing requirements as outlined at the authority's web site: sundhedsdatastyrelsen.dk/da/english/health_data_and_registers/research_services. Requests are normally processed within 3 to 6 months.

Analysis in the US-VA system was conducted under a waiver of informed consent with approval from the VA Boston Healthcare System Institutional Review Board. All VA data used in this study are available to any investigator upon relevant approvals through the VA Informatics and Computing Infrastructure (VINCI) (contact: VINCI@va.gov). Anyone wishing access to the data and use them for research will be required to meet research credentialing requirements as outlined by the VA office of Research and Development, which is expected to get processed at least within a half year.

Human research participants

Policy information about studies involving human research participants and Sex and Gender in Research.

Reporting on sex and gender	Statistics about sex are in Figure 2.
Population characteristics	Statistics about cohorts are in Figure 2.
Recruitment	There was no recruitment for this study but only access to existing databases. DNPR is a nation-wide registry and therefore includes data on the all patients having residence in Denmark. US-VA dataset includes data from all the veterans who access the veterans facilities.
Ethics oversight	The study has been approved by the Danish Health Data Authority (FSEID-00003092 and FSEID-00004491), the Danish Data Protection Agency (ref: SUND-2017-57) and GDPR record of processing activity (ref: 514-0255/18-3000). The VA Boston Healthcare System obtained internal permission from the Department of Veteran Affairs.

Note that full information on the approval of the study protocol must also be provided in the manuscript.

Field-specific reporting

Please select the one below that is the best fit for your research. If you are not sure, read the appropriate sections before making your selection.

☒ Life sciences ☐ Behavioural & social sciences ☐ Ecological, evolutionary & environmental sciences

For a reference copy of the document with all sections, see nature.com/documents/nr-reporting-summary-flat.pdf

Life sciences study design

All studies must disclose on these points even when the disclosure is negative.

Sample size	No sample size calculation was performed. The maximum number of patients was included in the analysis, after removal of incorrect or uninformative records.<br>DNPR dataset: 6,175,150 patients<br>US-VA dataset: 1,948,209 patients
Data exclusions	We excluded patients with insufficient followup (< 5 ICD codes), with discontinuous time history or temporary codes.
Replication	The models and hyperparameters were trained repeatedly on training and development sets and then evaluated on strictly withheld test sets, with careful attention to information separation between training and test sets.<br>The model training and evaluation were replicated on the US-VA dataset.
Randomization	Split into train, development and test sets was performed randomly on encrypted patient identifiers.
Blinding	In this context, random choice of test set is equivalent to blinding.

Reporting for specific materials, systems and methods

We require information from authors about some types of materials, experimental systems and methods used in many studies. Here, indicate whether each material, system or method listed is relevant to your study. If you are not sure if a list item applies to your research, read the appropriate section before selecting a response.

Materials & experimental systems

n/a	Involved in the study
☒	☐ Antibodies
☒	☐ Eukaryotic cell lines
☒	☐ Palaeontology and archaeology
☒	☐ Animals and other organisms
☒	☐ Clinical data
☒	☐ Dual use research of concern

Methods

n/a	Involved in the study
☒	☐ ChIP-seq
☒	☐ Flow cytometry
☒	☐ MRI-based neuroimaging
